# Inter- and trans-generational impacts of real-world PM_2.5_ exposure on male-specific primary hypogonadism

**DOI:** 10.1038/s41421-024-00657-0

**Published:** 2024-04-23

**Authors:** Xiaoyu Wei, Zhonghao Zhang, Yayun Gu, Rong Zhang, Jie Huang, Feng Li, Yuanlin He, Shuai Lu, Yifei Wu, Wentao Zeng, Xiaorui Liu, Chenzi Liu, Jinyi Liu, Lin Ao, Fuquan Shi, Qing Chen, Yuan Lin, Jiangbo Du, Guangfu Jin, Yankai Xia, Hongxia Ma, Yuxin Zheng, Ran Huo, Jia Cao, Hongbing Shen, Zhibin Hu

**Affiliations:** 1 State Key Laboratory of Reproductive Medicine and Offspring Health, Nanjing Medical University, Nanjing, Jiangsu China; 2 Department of Epidemiology, Center for Global Health, School of Public Health, Nanjing Medical University, Nanjing, Jiangsu China; 3 Key Lab of Medical Protection for Electromagnetic Radiation, Ministry of Education of China, Institute of Toxicology, College of Preventive Medicine, Army Medical University (Third Military Medical University), Chongqing, China; 4 Department of Toxicology, Hebei Medical University, Shijiazhuang, Hebei China; 5 Department of Occupational Health and Environmental Health, School of Public Health, Qingdao University, Qingdao, Shandong China

**Keywords:** Epigenetics, Methylation analysis

## Abstract

Exposure to PM_2.5_, a harmful type of air pollution, has been associated with compromised male reproductive health; however, it remains unclear whether such exposure can elicit transgenerational effects on male fertility. Here, we aim to examine the effect of paternal exposure to real-world PM_2.5_ on the reproductive health of male offspring. We have observed that paternal exposure to real-world PM_2.5_ can lead to transgenerational primary hypogonadism in a sex-selective manner, and we have also confirmed this phenotype by using an external model. Mechanically, we have identified small RNAs (sRNAs) that play a critical role in mediating these transgenerational effects. Specifically, miR6240 and piR016061, which are present in F0 PM sperm, regulate intergenerational transmission by targeting *Lhcgr* and *Nsd1*, respectively. We have also uncovered that piR033435 and piR006695 indirectly regulate F1 PM sperm methylation by binding to the 3′-untranslated region of *Tet1* mRNA. The reduced expression of *Tet1* resulted in hypermethylation of several testosterone synthesis genes, including *Lhcgr* and *Gnas*, impaired Leydig cell function and ultimately led to transgenerational primary hypogonadism. Our findings provide insights into the mechanisms underlying the transgenerational effects of paternal PM_2.5_ exposure on reproductive health, highlighting the crucial role played by sRNAs in mediating these effects. The findings underscore the significance of paternal pre-conception interventions in alleviating the adverse effects of environmental pollutants on reproductive health.

## Introduction

Infertility and poor semen quality are significant public health concerns, as increasing evidence links these conditions to environmental factors^1^. Among various environmental factors, particulate matter with a diameter of less than 2.5 micrometers (PM_2.5_) has garnered much attention in recent years due to its potential adverse effects on human health. Both epidemiological and animal studies have demonstrated an association between exposure to PM_2.5_ and a decreased sperm quality^2^, as well as an aberrant sperm morphology^3^. Parental exposure to environmental factors, such as diethylhexyl phthalate (DEHP)^4^, dichlorodiphenyltrichloroethane (DDT)^5^, and vinclozolin^6^, can have epigenetic effects on offspring phenotype that can persist across generations, sparking interest in exploring the impact of epigenetic effects on fertility^7^. However, the impact of paternal exposure to PM_2.5_ on offspring remains uncertain and requires further investigation^8,9^. Exposure to environmental toxins and stressors has been associated with transgenerational inheritance, where small RNA (sRNA) molecules, including microRNAs and piRNAs, along with changes in DNA methylation, are believed to play a role; however, their exact contributions are still under investigation. Additionally, the precise mechanism underlying the coding of these sRNAs through interactions with DNA methylation and other epigenetic factors is still being explored^10^.

Therefore, the present study investigated the transgenerational effects of paternal PM_2.5_ exposure on offspring’s reproductive health. We found that PM_2.5_ exposure led to the transgenerational effect of primary hypogonadism in male offspring. We also identified that sRNAs, especially piRNAs, played a crucial role in mediating this effect, shedding light on the intricate interplay between sRNAs and DNA methylation. These findings provide important insights into the mechanisms underlying the transgenerational effects of environmental pollutants on reproductive health and have significant implications for public health and environmental policymaking.

## Results

### Paternal PM_2.5_ exposure induces transgenerational transmission of primary male hypogonadism

**Fig. 1:**
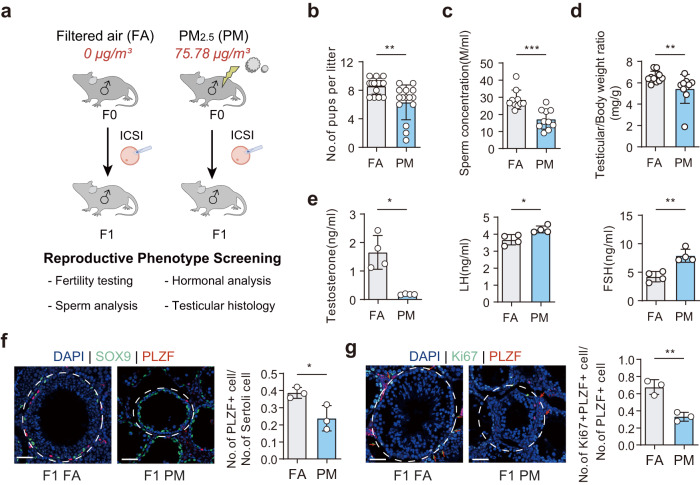
Paternal PM_2.5_ exposure induces intergenerational transmission of F1 primary male hypogonadism. **a** Schematic illustration of the experimental design. F0: Adult male mice were randomized to the FA and PM_2.5_ exposure. F1: Sperm from F0 groups was collected and used for ICSI to generate F1 offspring. **b** Number of pups per litter. *n* = 15 per group. **c** Sperm concentration analyzed by computer-assisted sperm analyzer (CASA) in adult F1 males at postnatal day (P)160–180. *n* = 10 per group. **d** Testicular/body weight ratio (mg/g) in adult F1 males (P160–P180). *n* = 10 per group. **e** Plasma testosterone, LH and FSH concentrations in adult males (P160–P180). *n* = 4 per group. **f** Left: Representative microscopy images of PLZF (red), SOX9 (green) and DAPI (blue) immunodetection on adult F1 seminiferous tubules (P160–P180). Scale bars, 50 μm. Right: Quantification of PLZF-positive/Sertoli cells (SOX9^+^) per tubule. **g** Left: Representative microscopy images of PLZF (red), Ki67 (green), and DAPI (blue) immunodetection on adult F1 seminiferous tubules (P160–P180). Scale bars, 50 μm. Red arrows represent PLZF-positive cells, and red/green arrows represent Ki67^+^PLZF^+^/PLZF^+^ cells. Right: Quantification of Ki67^+^PLZF^+^/PLZF^+^ cells per tubule. Data are represented as the mean ± SEM (black bar), and individual points represent biological replicates. For statistical analysis, *P* values were calculated by *t*-test (**b**, **f**, **g**). For normality or equal variance of samples failed, Mann–Whitney test was calculated (**c**–**e**). Statistical significance for all analyses was *P < 0.05; **P < 0.005; ***P < 0.0005.

We modeled a real-ambient and around-the-clock PM_2.5_ exposure system, as illustrated in Supplementary Fig. S1a. From the winter of 2019 to 2020, we conducted a murine PM_2.5_ exposure study for a total of 60 days in Shijiazhuang, China. Male C57BL/6 J mice were housed in either the filtered air (FA) control chambers or PM_2.5_ exposure chambers. After 60 days of PM_2.5_ exposure (0–60 days: 75.78 ± 6.41 μg/m^3^, PM group) (Supplementary Fig. S1b), mature sperm were collected. Intracytoplasmic sperm injection (ICSI) was used to generate FA-F1 and PM-F1 with normal oocytes (Fig. 1a).

PM-F1 male offspring exhibited major diagnostic criteria for male primary hypogonadism in humans^11,12^, including subfertility, reduced testicular weight, low sperm count and decreased testosterone levels, and increased levels of luteinizing hormone (LH) and follicle-stimulating hormone (FSH) (Fig. 1b–e; Supplementary Fig. S2a). PM-F1 male offspring also exhibited reduced sperm motility, compared with FA-F1 male offspring (Supplementary Fig. S2b). In comparison with FA-F1 male progeny, the examination for testicular histology of PM animals showed abnormalities that were associated with the hypogonadism phenotype, including the presence of thinner tubule wall heights, fewer germ cells, and more empty tubules (Supplementary Fig. S2c, d). To further characterize the germ cell phenotypes, we employed immunostaining with antibodies against the undifferentiated spermatogonia marker promyelocytic leukemia zinc-finger (PLZF) and Sertoli cell maker SRY-Box Transcription Factor 9 (SOX9) in the testes. We found that the number of PLZF-positive/Sertoli cells was significantly lower in PM-F1 testes than in FA-F1 testes (Fig. 1f). Additionally, sections of testes from FA-F1 and PM-F1 mice were co-immunostained with antibodies against PLZF and Ki67, a marker for cell proliferation. The ratio of Ki67 and PLZF-double positive cells to PLZF-positive cells (Ki67^+^PLZF^+^/PLZF^+^) in PM-F1 testes was significantly lower than that in FA-F1 testes (Fig. 1g), indicating that PM_2.5_ exposure in F0 fathers induced primary male hypogonadism and impaired maintenance of the population of undifferentiated spermatogonia in F1 male offspring.

**Fig. 2:**
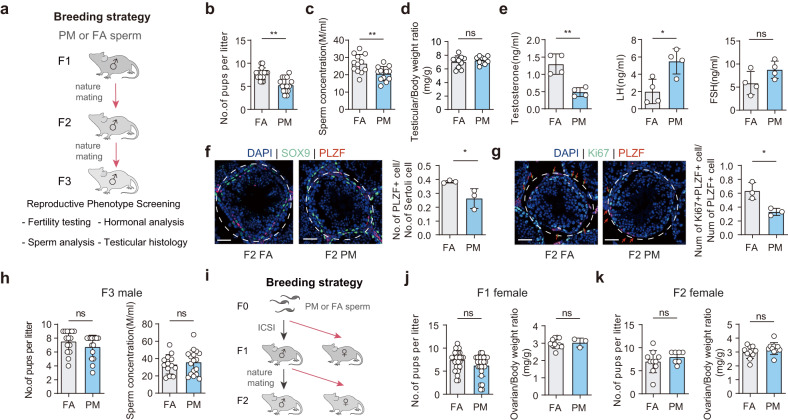
Paternal PM_2.5_ exposure induces transgenerational transmission of F2 primary male hypogonadism. **a** Breeding strategy to generate F2 and F3 male offspring. F2 and F3: Adult F1 males were mated with normal females, and F3 offspring were obtained in the same way. **b** Number of pups per litter of adult F2 males. *n* = 15 per group. **c** Sperm concentration in adult F2 males (P160–P180). *n* = 12 per group. **d** Testicular/body weight ratio (mg/g) in adult F2 males (P160–P180). *n* = 10 per group. **e** Plasma testosterone, LH and FSH concentrations in adult F2 males (P160–P180). *n* = 4 per group. **f** Left: Representative microscopy images of PLZF (red), SOX9 (green) and DAPI (blue) immunodetection on adult F2 seminiferous tubules (P160–P180). Scale bars, 50 μm. Right: Quantification of PLZF-positive/Sertoli cells (SOX9^+^) per tubule. **g** Left: Representative microscopy images of PLZF (red), Ki67 (green) and DAPI (blue) immunodetection on adult F2 seminiferous tubules (P160–P180). Scale bars, 50 μm. Red arrows represent PLZF-positive cells, and red/green arrows represent Ki67^+^PLZF^+^/PLZF^+^ cells. Right: Quantification of Ki67^+^PLZF^+^/PLZF^+^ cells per tubule. **h** Left: Number of pups per litter of adult F3 males. *n* = 15 per group. Right: Sperm concentration in adult F3 males (P160–P180) (FA: *n* = 13; PM: *n* = 17). **i** Breeding strategy to generate F1 and F2 female offspring. **j** Left: Number of pups per litter of adult F1 females. *n* = 27 per group. Right: Ovarian/body weight ratio in adult F1 females (P160–P180) (FA: *n* = 11; PM: *n* = 3). **k** Left: Number of pups per litter of adult F2 females (FA: *n* = 10; PM: *n* = 7). Right: Ovarian/body weight ratio in adult F2 females (P160–P180). *n* = 10 per group. Data are presented as the mean ± SEM. For statistical analysis, *P* values were calculated by *t*-test (**a**, **c**, **e**–**h**, **j**, and **k**). For normality or equal variance of samples failed, Mann–Whitney test was calculated (**b**, **d**). Statistical significance for all analyses was *P < 0.05; **P < 0.005.

To investigate the potential transmission of reproductive phenotype on subsequent generations, we employed a multi-generational breeding scheme, in which F1 PM males were mated with normal females to produce F2 offspring, and male descendants of F2 were subsequently mated with normal females to generate F3 offspring (Fig. 2a). Similar primary male hypogonadism features were observed in PM-F2 offspring, including a decreased fertility, low sperm count, low testosterone levels, and increased levels of LH (Fig. 2b, c, e). However, FSH levels in PM-F2 male offspring were comparable to those observed in FA-F2 offspring (Fig. 2e). PM-F2 exhibited less severe phenotypes based on testicular histopathology and morphology, with a comparable testicular/body weight ratio and sperm motility (Fig. 2d; Supplementary Fig. S3a) but more empty tubules in PM-F2 offspring (Supplementary Fig. S3b). Similarly, the PLZF-positive/Sertoli cell ratio and Ki67^+^PLZF^+^/PLZF^+^ ratio were lower in PM-F2 testes than those in FA testes (Fig. 2f, g). However, no phenotypic transmission was observed in F3 offspring, as there was no discernible difference in fertility, sperm count, sperm motility, testicular/body weight ratio, or testicular histology (Fig. 2h; Supplementary Fig. S3c–e). To assess the potential transmission of the reproductive phenotype in female offspring, we obtained F1 and F2 female offspring from F0 and F1 males with normal females (Fig. 2i). Importantly, our results indicated that female PM F1–F2 offspring did not exhibit any significant differences in litter size, ovarian/body weight ratio, the number of ovulated oocytes, or ovarian histopathology, relative to female FA F1–F2 offspring (Fig. 2j, k; Supplementary Fig. S3f–i).

### Independent verification of the transgenerational impact of PM_2.5_ exposure on primary male hypogonadism

Considering the intricacy of PM_2.5_ and the confounding effects introduced by assisted reproductive technologies, we created an additional PM_2.5_ exposure model in Tangshan (Supplementary Fig. S4a). Tangshan is approximately 368 kilometers away from Shijiazhuang and exhibits similar air pollution levels for comparison. Adult male mice were randomly assigned to either a chamber with FA or one with concentrated PM_2.5_ for 156 days (0–156 days: 83.75 ± 8.35 μg/m^3^, CAP group). These mice produced F1–F3 progeny by naturally mating with healthy female mice (Supplementary Fig. S4b). The male offspring of CAP-F1 exhibited primary hypogonadism characteristics, including reduced testicular/body weight ratio, low sperm count, reduced sperm motility, low testosterone levels, and increased levels of LH and FSH that were similar to those observed in PM-F1 (Supplementary Fig. S4c–e). Additionally, testicular histology revealed that CAP-F1 exhibited thinner tubule wall heights than FA-F1 (Supplementary Fig. S4l). Interestingly, transgenerational effects were observed in CAP-F2 male offspring, who also displayed hypogonadism characterized by low sperm concentration and testosterone levels as well as high LH levels. However, their testicular/body weight ratio, sperm motility, and FSH levels were comparable to those of FA-F2 offspring (Supplementary Fig. S4f–h). Furthermore, the testicular histopathology of CAP-F2 exhibited a higher degree of epithelial vacuolation compared to that observed in FA-F2 progeny (Supplementary Fig. S4m). Furthermore, the transmission of hypogonadism to CAP-F3 offspring was not observed (Supplementary Fig. S4i–k, n).

### Paternal exposure to PM_2.5_ impairs Leydig cell capacity for testosterone synthesis

To determine whether the male hypogonadism phenotype resulted from a reduction in Sertoli cell and Leydig cell numbers, sections were immunoassayed for SOX9, a marker for Sertoli cells, and insulin-like peptide 3 (INSL3), a marker for Leydig cells. When quantifying SOX9-positive cells per tubules, and tallying INSL3-positive cells per testis, no statistically significant differences were observed between F1–F2 mice from the FA and PM groups in terms of the number of Sertoli cells and Leydig cells (Supplementary Fig. S5a–d). We conducted single-cell transcriptome profiling of testicular cells (Supplementary Fig. S5e). Among Leydig and Sertoli cells, 180/200 and 495/463 differentially expressed genes were upregulated or downregulated, compared with FA-F1, respectively. In Leydig cells, pathway terms such as oxidative phosphorylation and androgen biosynthesis were suppressed, while cholesterol biosynthesis, GnRH signaling, etc., were increased (Supplementary Fig. S5f). Germ cell–Sertoli cell junction signaling and gap junction signaling, which are essential components of the blood–testis barrier and are regulated by testosterone, were inhibited in Sertoli cells. Meanwhile, mTOR signaling and EIF2 signaling were activated (Supplementary Fig. S5g). Furthermore, both single-cell transcriptome and real-time quantitative reverse transcription PCR (RT-qPCR) results revealed a downregulation of key enzymes involved in testosterone biosynthesis, specifically 3β- and steroid delta-isomerase 1 (*Hsd3b1*) and cytochrome P450 family 17 subfamily A member 1 (*Cyp17a1*), in PM-F1 (Supplementary Fig. S5h, i). Interestingly, PM-F2 testes also showed decreased expression of *Hsd3b1* and *Cyp17a1* (Supplementary Fig. S5j). This decline in protein levels of HSD3B1 and CYP17A1 was consistently observed in PM F1–F2 testes, as indicated by the Western blot (WB) and immunofluorescence results (Supplementary Fig. S6a–f). These findings suggest that the impairments observed in PM F1-F2 individuals may be attributed to compromised testosterone synthesis in Leydig cells.

### Sperm sRNAs induced paternal transmission of F1 male primary hypogonadism

**Fig. 3:**
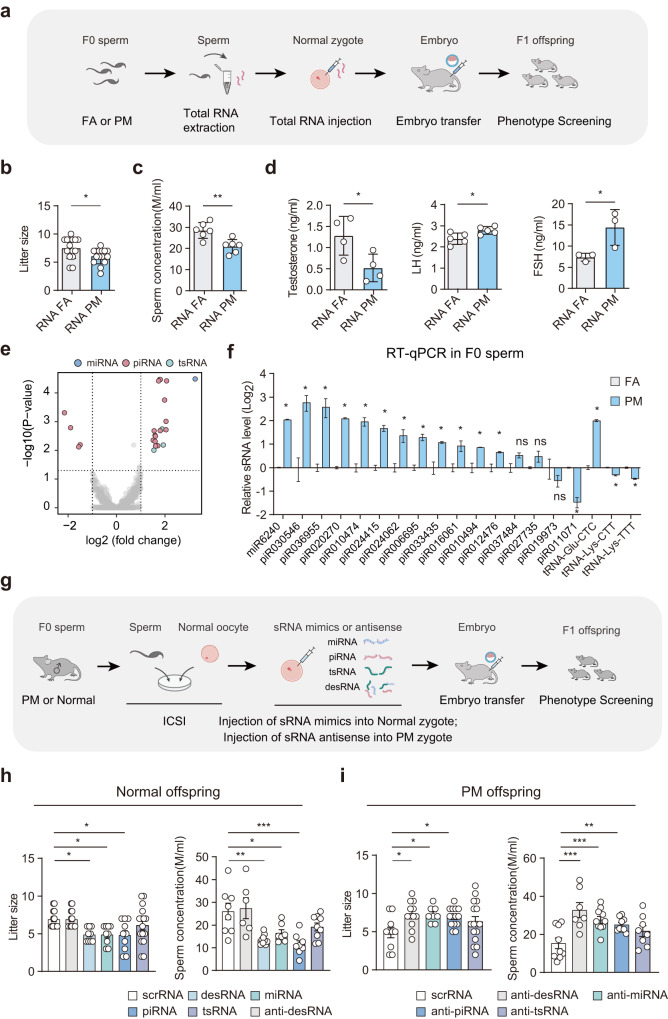
Sperm sRNAs conferred paternal transmission of male hypogonadism. **a** Schematic timeline of the ICSI offspring born to zygotes injected with sperm total RNA (RNA-FA and RNA-PM). Total RNA isolated from the sperm of FA-F0 and PM-F0 sperm was injected into normal zygotes to generate F1 offspring (RNA-FA and RNA-PM). The offspring were tested for fertility at 56 days of age. **b**, **c** Number of pups per litter (*n* = 15 per group) and sperm concentration (*n* = 6 per group) of RNA-FA and RNA-PM (P160–P180). **d** Plasma testosterone (*n* = 4 per group), LH (*n* = 4 per group) and FSH (*n* = 3 per group) concentrations of RNA-FA and RNA-PM offspring (P160–P180). **e** Volcano plot illustrating sperm sRNAs that were differentially expressed between the PM-F0 and FA-F0 groups (*n* = 3 per group). **f** RT-qPCR analysis of the expression of DESs in sperm derived from PM-F0 and FA-F0 (*n* = 3 per group). **g** Schematic timeline of the ICSI Normal offspring born to zygotes injected with different types of sRNA (miRNA, piRNA, tsRNA and desRNA), scrRNA or with anti-desRNA, PM offspring born to zygotes injected with sRNA antisense strands (anti-miRNA, anti-piRNA, anti-tsRNA and anti-desRNA) or with scrRNA. The offspring were tested for fertility at 56 days of age. **h** Number of pups per litter and sperm concentration of Normal offspring born to zygotes injected with scrRNA, anti-desRNA, miRNA, piRNA, tsRNA, desRNA (normal offspring: scrRNA *n* = 13, anti-desRNA *n* = 13, desRNA *n* = 9, miRNA *n* = 9, piRNA *n* = 10, tsRNA *n* = 16). **i** Number of pups per litter and sperm concentration of PM offspring born to zygotes injected with anti-miRNA, anti-piRNA, anti-tsRNA and anti-desRNA (PM offspring: scrRNA *n* = 10, anti-desRNA *n* = 12, miRNA *n* = 8, piRNA *n* = 14, tsRNA *n* = 16). Values are presented as the mean ± SEM. For statistical analysis, *P* values were calculated by *t*-test (**c**, **d**) or multiple *t*-test (**f**). For normality or equal variance of samples failed, Mann–Whitney test was calculated (**b**). Kruskal–Wallis one-way ANOVA followed by Dunnett’s correction was used (**h**, **i**). Statistical significance for all analyses was *P < 0.05; **P < 0.005; ***P < 0.0005.

To test whether the transgenerational effect were caused by sperm RNA. We then extracted total RNA from the sperm of FA and PM F0 mice and assessed whether injection of sperm RNAs at a standardized dose (approximately 10 sperm) could induce reproductive abnormalities in normal zygotes^13^. The embryos were subsequently implanted into surrogate mothers to produce offspring (RNA-FA and RNA-PM groups) (Fig. 3a). The litter size, sperm count, and testosterone levels of male offspring from the RNA-PM group were considerably lower than those of male offspring from the RNA-FA group, while the levels of LH and FSH were elevated in RNA-PM male offspring (Fig. 3b–d). Additionally, RNA-PM exhibited less severe phenotypes with a comparable testicular/body weight ratio and sperm motility but showed more empty tubules than the RNA-FA group (Supplementary Fig. S7a–c). Our findings suggest that sperm RNA of PM-F0 mice may have an intergenerational impact on male primary hypogonadism.

Using sRNA sequencing, we analyzed the sRNA profiles of sperm derived from FA-F0 and PM-F0 to determine whether sRNAs cause abnormalities in offspring. Analysis of differentially expressed sRNAs (DESs) using a stringent threshold (mean reads > 50, fold change > 1.5, and *P*_adj_ < 0.01) revealed that 1 miRNA, 20 piRNAs, and 3 tsRNAs were significantly altered in PM-F0 sperm, compared with those in FA-F0 sperm (Fig. 3e; Supplementary Table S1). We then validated the RNA deep sequencing results in 19 upregulated sRNAs by RT-qPCR. Thirteen DESs, including 1 miRNA, 11 piRNAs, and 1 tsRNA, were significantly increased in PM-F0 sperm (Fig. 3f).

We next synthesized sRNAs mimicking the 13 DESs in the sperm to investigate whether they can induce the transmission of reproductive phenotypes. A combination of 13 DESs, 3 different types of DESs or scrambled RNA (scrRNA) were injected separately into normal zygotes (desRNA, miRNA, piRNA, tsRNA and scrRNA) (Fig. 3g). Compared with scrRNA offspring, desRNA, miRNA and piRNA offspring showed a reduced litter size and a reduced sperm concentration, but tsRNA offspring did not (Fig. 3h). Furthermore, we observed that the injection of desRNA and piRNA into normal embryos led to a reduction in testosterone levels compared to the scrRNA group. Notably, LH and FSH levels displayed a modest increase in desRNA and piRNA offspring (Supplementary Fig. S7d). We also synthesized DES antisense strands to neutralize the effects of inherited sperm and injected them into PM or normal zygotes (Fig. 3g). scrRNA-PM exhibited considerable male hypogonadism traits, compared with scrRNA-Normal, whereas anti-desRNA-PM, anti-miRNA-PM, and anti-piRNA-PM showed a relatively larger litter size and sperm concentration, compared with those in scrRNA-PM, while anti-tsRNA-PM did not (Fig. 3i). As a control, anti-desRNA-normal did not display male hypogonadism, compared with scrRNA-normal, indicating that injection of DES antisense strands into zygotes alone had no obvious effect on offspring phenotypes. In PM embryos, the injection of anti-desRNA and anti-piRNA resulted in a significant elevation in testosterone levels when compared to the scrRNA group. Furthermore, LH and FSH levels exhibited conspicuous decreases in anti-desRNA and anti-piRNA offspring (Supplementary Fig. S7e). Taken together, these data showed that highly expressed miRNAs and piRNAs might serve as intergenerational carriers of male hypogonadism traits to the offspring.

### Sperm sRNAs downregulate the expression of genes involved in male gonadal development during early embryonic development

**Fig. 4:**
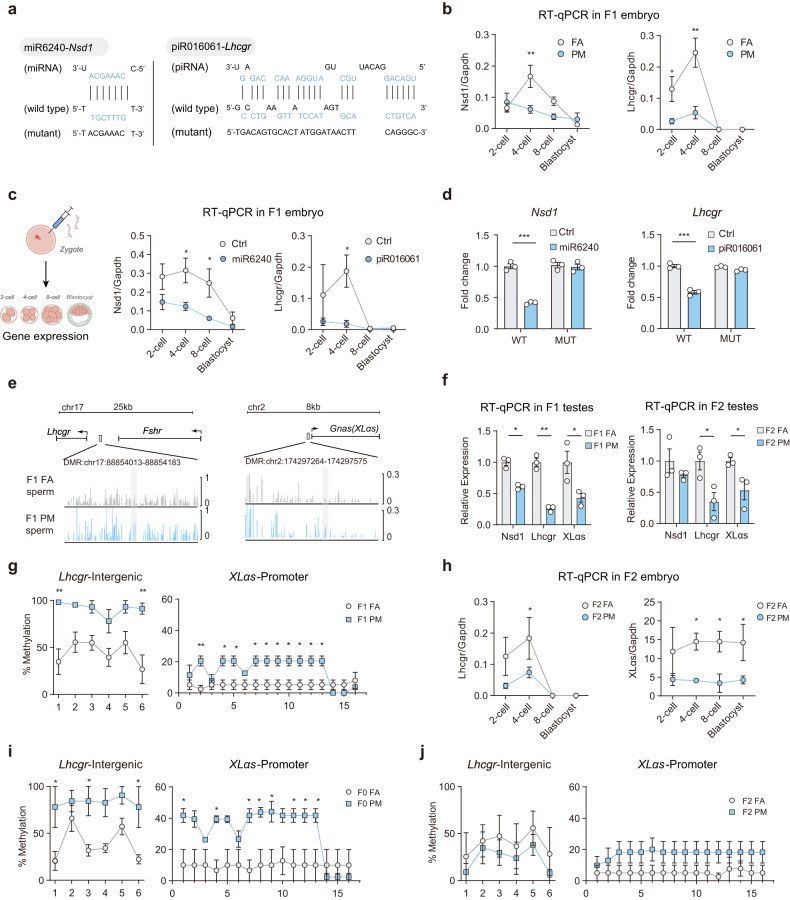
Sperm sRNAs and DMRs evoke transgenerational effects. **a** sRNA target prediction and schematic luciferase reporter constructs. piR016061 targets the 3′-UTR of *Lhcgr*, and miR6240 targets the 3′UTR of *Nsd1*. A mutant construct was made by replacing nucleotides in the sRNA binding site of the target gene 3′UTR. **b** Line chart of the relative expression levels of *Nsd1* and *Lhcgr* in early embryos. Zygotes derived from FA-F0 or PM-F0 were cultured, and the gene transcriptional changes were assessed across early embryonic stages (*n* = 3). *Gapdh*, glyceraldehyde-3-phosphate dehydrogenase. **c** Line chart of the relative expression levels of *Lhcgr* and *Nsd1* in early embryos. Zygotes were injected with Ctrl (scrRNA), miR6240 and piR016061 mimics, and the gene transcriptional changes were assessed across early embryonic stages (*n* = 3). **d** Luciferase reporter assay of the *Lhcgr* (left) and *Nsd1* (right) 3′-UTR reporters in 293T cells 48 h after transfection. WT: wild type; MUT: mutant; Ctrl: scrambled negative control. **e** Methylation profile of the *Lhcgr* and *XLαs* genes in the sperm of PM vs FA in F1 mice. Vertical bars (FA, gray; PM, blue) above the horizontal line indicate the methylation level at individual CpGs. **f** RT-qPCR analysis of the expression of *Lhcgr*, *Nsd1* and *XLas* in F1–F2 testes derived from FA vs PM (*n* = 3 per group). **h** Line chart of the relative expression levels of *Lhcgr* and *XLαs* in early embryos. Zygotes derived from FA-F1 or PM-F1 were cultured, and the gene transcriptional changes were assessed across early embryonic stages (*n* = 3). Bisulfite sequencing PCR analysis of the *Lhcgr* intergenic region and *XLαs* promoter in F1, F0 and F2 sperm from FA and PM (*n* = 3) (**g**, **i**, **j**). Values are presented as the mean ± SEM. For statistical analysis, *P* values were calculated by two-way ANOVA followed by Fisher’s LSD test (**b**, **c**, **g**–**j**) or multiple *t*-test (**d**, **f**). Statistical significance for all analyses was *P < 0.05; **P < 0.005; ***P < 0.0005.

We also performed RT-qPCR to examine the expression levels of highly expressed miRNA and piRNAs in F1 zygotes and observed a significant increase in five DESs in PM-F1 zygotes (Supplementary Fig. S8a). Subsequently, we utilized DIANA Tools (http://diana.imis.athena-innovation.gr/DianaTools/index.php) and TargetScan (www.targetscan.org/vert_72/) databases for the target prediction analysis, which identified a total of 400 potential target genes (Supplementary Table S2). Many of the 400 genes were shown to be involved in the control of male gonadal development through literature mining (e.g., cAMP signaling pathway^14^, cellular response to hormone stimulus^15^). Among these putative sRNA-target pairs, piR016061-Lhcgr and miR6240-Nsd1 were selected as representative and specifically characterized (Fig. 4a). The expression of *Nsd1* increased dramatically at the four-cell stage in FA-F1 embryos, whereas in PM-F1 embryos, the elevation of *Nsd1* was diminished (Fig. 4b). *Lhcgr* expression ranged between 2 and 4 cells in FA-F1 embryos; however, it was reduced in PM-F1 embryos (Fig. 4b). We next demonstrated that the expressions of *Lhcgr* and *Nsd1* were reduced in embryos injected with miR6240 or piR016061, compared with the control group (Fig. 4c). Furthermore, the sRNA was found to directly bind to the 3′ untranslated regions (UTRs) of the *Lhcgr* and *Nsd1*, as confirmed by the luciferase reporter assay (Fig. 4d). These findings suggested that an overabundance of sRNA may trigger intergenerational effects by reducing *Lhcgr* and *Nsd1* expressions.

### Sperm differentially methylated regions (DMRs) evoke transgenerational effects on F2 male offspring

Although we observed a transgenerational impact on male offspring of PM exposure, it is unlikely that sRNAs may have long-term effects on transgenerational phenotypes in mammals due to the absence of a recognized RNA-dependent RNA polymerase, which is present in many model organisms, such as plants and worms, playing a role in amplifying sRNA signals^8,16^. As expected, no significant alterations of the 13 DESs in PM-F0 sperm were observed in PM-F1 sperm (Supplementary Fig. S8b).

We subsequently employed whole-genome bisulfite sequencing to investigate the DNA methylation patterns in F1 sperm of PM and FA mice. The analysis revealed a significant elevation in methylation levels in PM sperm compared to those in FA sperm (Supplementary Fig. S8c). Considering the predominant phenotype characterized by a decline in testosterone and reduced expression of enzymes associated with testosterone synthesis, we have focused on the methylation status of genes related to testosterone synthesis pathway. In this context, we discerned hypermethylated DMRs within the intergenic region of *Lhcgr* and the promoter region of *Gnas (XLαs)* in PM-F1 sperm (Fig. 4e). Notably, these genes serve as receptors for LH and have the capability to modulate the expression of testosterone synthesis enzymes such as CYP17A1 and HSD3B1. Additionally, the expressions of *Lhcgr* and *XLαs* in early-stage embryos of F2 PM were lower than those in F2 FA embryos (Fig. 4h). Bisulfite sequencing PCR revealed the hypermethylation of these two DMRs in PM F0-F1 sperm but not in F2 (Fig. 4i, j). Furthermore, the decreased expressions of *Lhcgr* and *XLαs* were sustained in adult testes of PM F1-F2 (Fig. 4f). WB and immunofluorescence results similarly indicated a reduction in protein levels in the PM testis compared to the FA testis (Supplementary Fig. S6a–f). These findings suggested that DNA methylation changes in PM sperm may be inheritable and impact the expression of genes associated with male primary hypogonadism in subsequent generations.

### Increased piRNAs expression in F0 sperm contributes to hypermethylation of *Lhcgr* and *Gnas* in F1 sperm

**Fig. 5:**
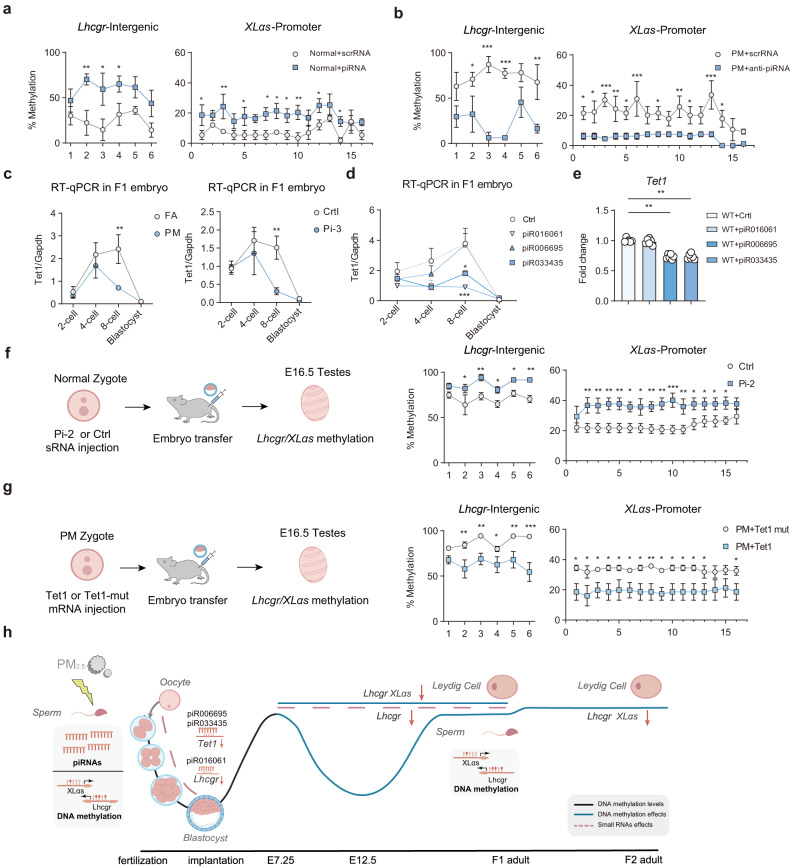
Increased piRNAs expression in F0 sperm contributes to hypermethylation of *Lhcgr* and *Gnas* in F1 sperm. **a** Bisulfite sequencing PCR analysis of the *Lhcgr* intergenic region and *XLαs* promoter in F1 sperm from normal offspring born to zygotes injected with piRNA or scrRNA (*n* = 3). **b** Bisulfite sequencing PCR analysis of the *Lhcgr* intergenic region and *XLαs* promoter in F1 sperm from PM offspring born to zygotes injected with anti-piRNA or scrRNA. (*n* = 3). **c** Right: Line chart of the relative expression levels of *Tet1* in early embryos. Zygotes derived from FA-F1 or PM-F1 were cultured, and the gene transcriptional changes were assessed across early embryonic stages (*n* = 3). Left: Line chart of the relative expression levels of *Tet1* in early embryos. Zygotes were injected with Ctrl (scrRNA) or Pi-3 (piR033435, piR016061, and piR006695 mimics), the gene transcriptional changes were assessed across early embryonic stages (*n* = 3). **d** Line chart of the relative expression levels of *Tet1* in early embryos. Zygotes were injected with Ctrl (scrRNA), piR033435, piR016061, and piR006695 mimics, and the gene transcriptional changes were assessed across early embryonic stages (*n* = 3). **e** Luciferase reporter assay of the *Tet1* 3′-UTR reporters in 293T cells 48 h after co-transfection with Ctrl (scrRNA), piR033435, piR016061 and piR006695 mimics. WT: wild type; Ctrl: scrambled negative control. **f** Bisulfite sequencing PCR analysis of the *Lhcgr* intergenic region and *XLas* promoter in E16.5 testes from normal offspring born to zygotes injected with Pi-2 (piR033435 and piR006695) or Ctrl (scrRNA) (*n* = 3). **g** Bisulfite sequencing PCR analysis of the *Lhcgr* intergenic region and *XLas* promoter in E16.5 testes from PM offspring born to zygotes injected with *Tet1* mRNA (*n* = 3). **h** Model illustrating the transgenerational effect of PM_2.5_ exposure on male fertility. Our study revealed that paternal PM_2.5_ exposure results in a transgenerational effect on male offspring, with F1 and F2 male offspring showing traits of male hypogonadism, while female offspring remain unaffected. Our study found the impact of PM_2.5_ exposure on sRNA changes, with an abnormal increase in piRNAs levels and an increase in DNA methylation. While we did not observe piRNAs maintenance in F1 sperm, we did find that DNA methylation levels may have a lasting effect. Interestingly, piRNA molecules were found to have transgenerational and intergenerational effects through regulation of target genes. One sRNA molecule, piR016061, was found to target *Lhcgr*, leading to functional changes in Leydig cells and decreased testosterone production in F1 offspring. Furthermore, high levels of DNA methylation in *Lhcgr* and *Gnas* in F0 generation mice may also contribute to Leydig cell dysfunction. Additionally, we found that other piRNAs, such as piR006695 and piR033435, regulate the methylation levels of *Lhcgr* and *Gnas* in F1 male germ cells by targeting *Tet1*, leading to transgenerational effects on Leydig cell function in F2 offspring. Values are presented as the mean ± SEM. For statistical analysis, *P* values were calculated by two-way ANOVA followed by uncorrected Fisher’s LSD test (**a**–**c**, **f**, **g**) or one-way ANOVA followed by Dunnett’s multiple comparisons test (**d**, **e**).

To address whether the paternally increased sRNAs cause DNA methylation alterations, we measured the methylation level of F1 sperm from mice injected with synthesized sRNAs (Supplementary Fig. S8d). Compared with scrRNA-Normal offspring’s sperm, piRNA offspring’s sperm exhibited elevated methylation levels in *Lhcgr* and *XLαs*, but miRNA offspring’s sperm did not (Fig. 5a; Supplementary Fig. S8e). When synthesized DES antisense strands were injected into PM zygotes, the injection of anti-piRNAs showed reversed methylation levels, compared with that of scrRNA, but not anti-miRNA (Fig. 5b; Supplementary Fig. S8f).

DNA demethylation in primordial germ cells goes through three stages: loss of bulk DNA methylation in a Tet-independent manner; oxidation of remaining 5mC to 5hmC by Tet1 and potentially Tet2; and loss of 5hmC through replication-dependent passive dilution^17^. Based on these, we formulated the hypothesis that piRNAs may play a role in regulating Tets. We used miRanda v3.3a to identify potential piRNA targets within the 3′-UTRs of *Tet1* and *Tet2*, and we found that piR033435, piR006695, and piR016061 may act on *Tet1* and *Tet2*. RT-qPCR analysis revealed a reduction in *Tet1* expression during the early stages of PM-F1 embryos but not *Tet2* (Fig. 5c; Supplementary Fig. S8g). Further investigation through zygote injection with Pi-3 (i.e., piR033435, piR006695, and piR016061 mimics) confirmed that Pi-3 reduced *Tet1* levels but not *Tet2* (Fig. 5c; Supplementary Fig. S8g). To evaluate the relevance of these piRNAs to *Tet1* expression, we generated a luciferase reporter construct containing the *Tet1* 3′-UTR. Co-transfection of 293T cells with Pi-3 reduced the luciferase activity of the *Tet1* 3′-UTR construct (Supplementary Fig. S8h). Notably, we identified that piR033435 and piR006695, rather than piR016061, can regulate *Tet1* levels (Fig. 5d, e). As we found the same phenotype in the external model, so we tested whether these piRNAs were also increased in the sperm of CAP-F0 group. Similarly, we observed a similar increase in piR033435 and piR006695 expressions in the sperm of the CAP-F0 group, indicating their potential role in modulating DNA methylation in germ cells (Supplementary Fig. S8i). The selection process of piRNAs for potential effects and the prediction of target genes for effective piRNAs are shown in Supplementary Fig. S9.

Next, we examined whether the regulation of *Tet1* might lead to changes in DNA methylation in the male germline. As previously stated, global methylation of F1 sperm DNA in the PM group was significantly increased, compared with FA (Supplementary Fig. S7c). We then examined the DNA methylation status of *Lhcgr* and *XLαs* in testes from mice injected with Pi-2 (piR033435 and piR006695 mimics), and testes from Pi-2 mice exhibited higher methylation levels, compared with controls (Fig. 5f). Moreover, the restoration of *Tet1* mRNA in PM zygotes resulted in a significant reduction in hypermethylation of *Lhcgr* and *XLαs* in testes (Fig. 5g). Taken together, these findings suggest that the observed increase in DNA methylation levels in PM-F1 sperm may potentially contribute to transgenerational effects on male primary hypogonadism and that this increase in DNA methylation could be attributed to an elevation in the abundance of specific piRNAs (Fig. 5h; Supplementary Fig. S10).

## Discussion

Epigenetic modifications induced by environmental pollutants have been implicated in the decline of male fertility. To investigate the transgenerational effects of real-world PM_2.5_ exposure, we used a model to simulate such exposure and validated our findings through an external model. Our study has revealed that paternal PM_2.5_ exposure can have a transgenerational impact on male offspring, with both F1 and F2 male offspring displaying male hypogonadism traits, while female offspring remain unaffected. Interestingly, we observed an abnormal increase in piRNA levels and DNA methylation in the sperm of F0 following PM_2.5_ exposure. Although piRNA maintenance was not observed in F1 sperm, DNA methylation levels were found to have a lasting impact. Notably, piRNA molecules were found to have intergenerational and transgenerational effects through the regulation of target genes. One sRNA molecule, piR016061, was found to target *Lhcgr*, resulting in a reduction in the expression of *Cyp17a1* and *Hsd3b1*, which are downstream of Lhcgr. This led to functional changes in Leydig cells and decreased testosterone production in F1 offspring. Furthermore, high levels of DNA methylation in *Lhcgr* and *XLαs* in F0 generation mice may also contribute to Leydig cell dysfunction. Additionally, we have found that other piRNAs, such as piR006695 and piR033435, regulate the methylation levels of *Lhcgr* and *XLαs* in F1 male germ cells by targeting *Tet1*, leading to transgenerational effects on Leydig cell function in F2 offspring. Overall, the present study provides important insights into the transgenerational effects of PM_2.5_ exposure and its subsequent impact on male fertility.

The conventional approach to chemical risk assessment, which focuses on an individual or a few chemicals in high doses and relies on strict regulations and avoidance of exposure sources, has proven to be effective in protecting humans from diseases caused by certain hazards^18^. For example, endocrine-disrupting chemicals (EDCs), such as bisphenol A^19^, di-n-butyl phthalate^20^, and benzo(a)pyrene^21^ can generate paternal transgenerational inheritance of epigenetic alterations, leading to poor reproductive outcomes in offspring. However, environmental exposure to toxins rarely arises from a single source or chemical substance. Instead, it typically results from long-term exposure to mixtures of chemicals with additive, overlapping, or complementary mechanisms of action. Therefore, it is crucial to investigate the effects of chemical mixtures in a real-world context to better understand their potential impacts on human health. Our findings indicate that long-term PM_2.5_ can pose a significant risk to reproductive health. These findings underscore the need to re-evaluate traditional risk assessment methods and consider the potential health impacts of chemical mixtures.

Mammalian sperm RNA is increasingly recognized as a critical source of paternal hereditary information^22^. Environmental factors, such as an unhealthy diet, mental stresses, and toxin exposure, can reshape the sperm RNA signature and induce offspring phenotypes. Although piRNAs in *C. elegans* and *D. melanogaster* play a role in germline-inherited epigenetic silencing^23^, their function in mammals remains uncertain. In the present study, we have found that sRNAs, such as microRNA and piRNAs, highly expressed in PM_2.5_-exposed sperm can lead to a reduced sperm count and litter size in offspring. Moreover, neutralization of these sRNAs by antisense strands partly rescued male hypogonadism phenotypes. We have also revealed that sperm piRNAs are sensitive to environmental factors and can serve as transgenerational carriers of epigenetic information, affecting F2 phenotypes by regulating *Tet1* expression. While this study has provided initial insights into the intricate interplay between sRNA and DNA methylation, further research is essential to ascertain whether piRNA can facilitate de novo DNA methylation in mature sperm. Furthermore, previous studies have suggested that long RNA^24^ and rsRNA^25,26^ may mediate transgenerational effects. In this study, we analyzed the differential expression of rsRNA through sRNA sequencing data, identifying 27 rsRNAs that exhibited significant changes in PM-exposed sperm relative to FA-exposed sperm (data not shown). Subsequently, we selected the top 15 upregulated rsRNAs for further validation, and 3 of them were found to maintain elevated expression in PM F1 hybrids. This suggests that rsRNAs are susceptible to PM_2.5_ exposure. It is worth noting that there exist several sRNA sequencing methods, such as PANDORA-seq^27^ or CPA-seq^28^, which are capable of identifying a wider range of tsRNAs and rsRNAs. Therefore, the conventional sRNA-seq method employed in this study may have limitations in detecting functional sRNAs. Furthermore, this study primarily focuses on linear complementarity-based mechanisms of sRNAs; however, recent research suggests that tsRNAs or rsRNAs may exert their functions through 3D structures^29^. Hence, further investigation is required to elucidate the role of RNA in epigenetics.

Recent research has suggested that exposure to PM_2.5_ may have endocrine-disrupting properties that could potentially impact male reproductive health^3^. It is worth noting that environmental compounds, such as EDCs, are known to cause transgenerational effects in a sex-specific manner^7^. The findings of the present study align with this knowledge, as it shows that transgenerational effects of PM_2.5_ exposure are limited to male offspring. It is well-established that EDCs can bind to estrogen and testosterone receptors, potentially eliciting similar effects to natural ligands and thereby leading to gender-specific transgenerational impacts on offspring^30^. Therefore, it is essential to identify the critical factors that may contribute to the observed sex-specific transgenerational effects of PM_2.5_ exposure. The present study has identified Lhcgr as one such factor, which plays a pivotal role in male fetal development by binding to placental chorionic gonadotropin and producing testosterone^31^. Testosterone is an essential hormone for male gonad development, and the inactivity of Lhcgr results in male pseudohermaphroditism^32^. However, LH or steroid hormones are not necessary for female fetal sex differentiation, as observed in the genes of women with LHR mutation, who display normal female development of internal and external genitals and normal pubertal maturation^33^. These findings may partially explain the transgenerational sex disparities in PM_2.5_-exposed subjects. Additionally, in the clinical management of primary hypogonadism, the established approach involves testosterone replacement therapy^11^. Unfortunately, this study did not employ testosterone supplementation, leaving the potential restorative effects of testosterone on the phenotype of offspring undetermined. Addressing this issue would necessitate further experimental endeavors.

To the best of our knowledge, the present study is the first to report a transgenerational effect of primary hypogonadism in male offspring, resulting from paternal exposure to PM_2.5_. Given the high prevalence of male hypogonadism, which is often underdiagnosed and undertreated, these findings have significant implications. Moreover, with PM_2.5_ management on the decline and the potential for parents to be exposed to high levels of this type of pollutants, an increased awareness and monitoring of male reproductive health is urgently needed. The insights gained from the present study on epigenetic modifications underlying susceptibility to reduced fertility highlight the urgent need for developing prevention and treatment strategies to mitigate the impact of environmental pollutants on male reproductive health. As such, the present study has the potential to catalyze efforts to better understand and address the growing threat that environmental pollutants pose to male fertility.

## Materials and methods

### Mice and ethics statement

C57BL/6 J mice were acquired from Vital River (Charles River) and Jiangsu Laboratory Animal Center of Nanjing Medical University (Nanjing, China), and all mice were maintained under 14-h/10-h light-dark cycles (dark from 20:00 to 06:00). All studies were authorized by the Third Medical University of Laboratory Animal Welfare (AMUWEC20193803) and the Ethics Care Committee and the Nanjing Medical University Institutional Animal Care and Research Committee (IACUC-2001004).

### Animal study design

In the present study, our objective was to investigate the intergenerational and transgenerational effects of paternal exposure to PM_2.5_ on offspring. Two mouse models were established in two separate places located in Hebei Province, that is, Shijiazhuang and Tangshan, 368 kilometers apart.

### PM_2.5_ mouse model and assisted reproductive F1 offspring

In Shijiazhuang, we designed a PM exposure system by remodeling a conventional individually ventilated cages (IVC) system as described previously (Supplementary Fig. S1a)^34^. The humidity was 40%–60%, and the temperature was 20–25 °C in the cages with a 12-h light/dark cycle for a total of 60 days (November 2019–January 2020).

C57BL/6 J males aged 8 weeks were randomly divided into three groups: the first group (PM-F0) was exposed to unfiltered air by real-time ambient particulate matter exposure chambers for 60 days, the second group (FA-F0) was subjected to filtered air for 60 days. Immediately following PM_2.5_ exposure, sperm from these three groups were collected and used for ICSI to produce F1 offspring (FA-F1 and PM-F1) (Fig. 1a), while the remaining sperm were used for sRNA sequencing. FA-F1 and PM-F1 were screened for mouse fertility of male and female mice; testicular and ovarian pathologies; sperm quality; serum FSH, LH, and testosterone levels; and the population of undifferentiated spermatogonia. F2 offspring were produced by mating F1 males with normal females, and F3 offspring were produced in the same manner. Then, ICSI techniques were utilized to causally examine whether male subfertility traits in fathers are transferred to offspring via sperm, sperm total RNA, sperm desRNA, sperm miRNA, sperm tsRNA and sperm piRNA. The fertility of male mice; testicular pathology; sperm quality; serum FSH, LH, and testosterone levels were assessed in the same manner as described previously. Whole-genome methylation of F1 sperm was performed to unveil the mechanism of the transgenerational effects.

### Concentrated PM_2.5_ mouse model and naturally mated F1 offspring

C57BL/6 J males aged 8 weeks were randomly divided into two groups. The FA group was given ambient air that had been filtered by a highly efficient particulate air filter, whereas the CAP group was given concentrated ambient PM_2.5_ with a PM_2.5_ concentration enrichment system (Beijing Huironghe Technology Co., Ltd.) (Supplementary Fig. S4a). The duration of the exposure protocol was 156 days (November 2019–April 2020). To ensure that the transgenerational effects were from the male lineage, we used unrelated female breeders who were not exposed to PM_2.5_. Briefly, after the last exposure, mice of each group were naturally mated with normal female mice to generate the F1 offspring. F2 offspring were produced by mating F1 males with normal females, and F3 offspring were produced in the same way (Supplementary Fig. S4b). None of the F1–F3 offspring were directly exposed to PM_2.5_. Reproductive parameters were evaluated for each generation of adult males, including testicular pathology, sperm quality, and reproductive hormones.

### Oocyte and zygote collection

Embryo collection and transfer were performed as stated previously. In brief, 4-week-old virgin female mice served as oocyte donors for superovulation, which was induced by intraperitoneal injection of 5 IU pregnant mare serum gonadotrophin 48 h following intraperitoneal injection of human chorionic gonadotropin (hCG). Oocytes were collected 12–18 h after hCG administration, and zygotes were collected from successfully mated female mice.

### Microinjection of sperm heads, sperm RNA, sperm synthetic mimic, and antisense sRNA, and transfer of embryos

Sperm total RNA was isolated using the TRIzol Reagent (Invitrogen, Cat# 15596018). Synthetic sRNA mimics, antisense, and scrRNA were purchased from RiboBio Co., Ltd. The sequences of synthetic sRNA mimics and antisense strands are listed in Supplementary Tables S3 and S4.

MII (first polar body present) oocytes were used to perform ICSI, and fertilization was confirmed by the presence of two pronuclei. Total RNA and synthetic sRNA were adjusted to a concentration of 2 ng/μL (each sRNA mimic or antisense strand was 0.125 ng/μL). All RNAs were microinjected into zygotes of the C57BL/6 J background using a Leica microinjection system. The zygotes were then cultured in M16 (Sigma-Aldrich) at 37 °C in 5% CO_2_. The two-cell embryos were transferred to the oviduct of a surrogate mother of the Institute of Cancer Research background. The offspring of each group were screened for the fertility of male mice and sperm quality.

### Assessment of phenotype, fertility, and sperm concentration

Two females were placed overnight in a male’s cage, and each morning, the females were inspected for the presence of a vaginal plug. Females were relocated to individual cages on the day that a vaginal plug was discovered (GD 0). On the 18th day of pregnancy, females were checked for birth, and the number of pups per pairing was calculated. Mouse sperm samples were collected from the cauda epididymis of adult male mice and incubated at 37 °C for 5 min in human tubal fluid (HTF) medium (Millipore, Cat# MR-070-D) supplemented with 10% fetal bovine serum. A CASA (USA) was utilized for sperm counting and motility analysis.

### Testosterone, LH and FSH assessment

LH, FSH and testosterone were measured in the serum of F1–F3 male offspring using a mouse LH ELISA Kit (S-type) (FUJIFILM Wako Shiba Yagi Corporation, Cat# 630-23929), a mouse FSH ELISA kit (Abbexa, Cat# abx154038), and a DetectX® Testosterone ELISA Kit (Arbor Assays Inc, Cat# K032-H1), respectively. Serum samples from a concentrated PM_2.5_ mouse model were detected by NanJing JianCheng Bioengineering Institute.

### RT-qPCR analyses of mRNA and sRNA

Total RNA was isolated from tissues and sperm using the TRIzol Reagent according to the manufacturer’s instructions. RT-qPCR was performed using ChamQ Universal SYBR qPCR Master Mix (Vazyme) and a Q7 real-time PCR System (Applied Biosystems) according to the manufacturer’s instructions. *Gapdh* was employed as an endogenous control, and the threshold cycle (Ct) for each test was established.

For sRNA analyses, 100 ng of total RNA was reverse transcribed to cDNA using a PrimeScript™ RT Reagent Kit (Takara, Japan) and stem-loop RT primer (Tsingke Biotechnology Co., Ltd.). RT-qPCR was performed using ChamQ Universal SYBR qPCR Master Mix (Vazyme) and a Q7 real-time PCR System (Applied Biosystems). The relative expression of sRNA was normalized to U6 snRNA. The employed primers are listed in Supplementary Tables S5 and S6.

### Hematoxylin and eosin (H&E), Periodic acid-Schiff (PAS), immunofluorescence, and immunohistochemistry (IHC) staining

For histology and immunostaining, the testis and epididymis of mice were fixed with Davidson’s fixative or 4% paraformaldehyde (PFA) and then dehydrated. Sectioned (6 µm) paraffin-embedded specimens were mounted on glass slides.

For immunofluorescence analysis, after deparaffinization, rehydration, and antigen retrieval in 0.01% sodium citrate buffer (pH 6.0) (Servicebio), the tissue was permeabilized for 30 min with PBS containing 0.1% Triton X-100. Then, the tissue sections were blocked with 5% bovine serum albumin for 2 h at room temperature. Sections were incubated overnight at 4 °C with the following primary antibodies: rabbit anti-SOX9 (Millipore, Cat# AB5535), goat anti-PLZF (R&D Systems, Cat# AF2944), rabbit anti-CYP17A1(Abcam, Cat# ab125022), rabbit anti-HSD3B1(Invitrogen, Cat# MA5-42697), rabbit anti-LHCGR (Invitrogen, Cat# PA5-115508), rabbit anti-XLαs (Arigo, Cat# ARG43266) and rat anti-Ki67 (Invitrogen, Cat# 14-5698-82). The primary antibody was washed with PBS with Tween 20 (PBST), and then secondary antibodies conjugated to Alexa Fluor 488 (Invitrogen, Cat# A27012), Alexa Fluor 555 (Invitrogen, Cat# A31570), or Cy3-conjugated ChromPure Goat IgG (Jackson ImmunoResearch, Cat# AB_2337004) were added and incubated for 2 h at room temperature. After being washed in PBS, the slides were stained with DAPI (Servicebio, China).

For IHC analysis, IHC-based labeling was performed after deparaffinization using a Biotin-Streptavidin HRP Detection System (ZSGB-BIO, Cat# SP-9001 and Cat# SP-9002) with mouse anti-INSL3 (ABclone, Cat# A5728) and rabbit anti-SOX9. All slides were counterstained with hematoxylin.

Prior to staining with H&E and PAS, the tissue sections underwent deparaffinization and rehydration. Hematoxylin and eosin were used to stain the H&E slides. For PAS staining, the tissue sections were first oxidized in a 0.5% periodic acid solution at 37 °C for 15 min, followed by washing with distilled water and placement in Schiff reagent for 25 min. After counterstaining with hematoxylin for 2 min, the slide was shaken twice in 1% HCl, dehydrated, and mounted. Bright-field images were obtained with a microscope (Pannoramic SCAN150, Budapest, Hungary), and fluorescent images were captured with a confocal microscope (LSM 800, Carl Zeiss). Briefly, as previously described, all Stage VII tubules (defined by the presence of preleptotene spermatocytes, 20 per section) were detected. Assessment was performed of their diameters, wall heights, and numbers of Sertoli cells and germ cells, including spermatogonia, spermatocytes, and spermatids. The abundance of these cells was expressed as the corrected number per seminiferous tubule or Sertoli cells^35^. The Leydig cells were counted as described in ref. ^36^.

### Protein extraction and WB analysis

Tissues were lysed using the mammalian protein extraction reagent RIPA (Beyotime). The protein lysates were cleared by centrifuging, and the concentrations were qualified using the BCA protein assay kit (Beyotime). Then the lysates were combined with 5× Sample Loading Buffer (Beyotime) followed by denatured at 95 °C for 5 min. For the Western blotting, 20 μg of the protein were separated by 4%–12% SDS-PAGE and transferred to a 0.45-μm PVDF membrane (Millipore). Membranes were then blocked in 5% BSA and incubated with rabbit anti-CYP17A1(1:3000, Abcam, Cat# ab125022), rabbit anti-HSD3B1(1:2000, Invitrogen, Cat# MA5-42697), rabbit anti-LHCGR (1:2000, Invitrogen, Cat# PA5-115508), rabbit anti-XLαs (1:1000, Arigo, Cat# ARG43266) and rabbit anti-GAPDH (1:3000, Cell Signaling Technology, Cat# 2118) respectively. Immunoreactive proteins were visualized using a molecular imager (Bio-Rad).

### Histological analysis of ovaries

Ovaries were fixed in PFA overnight and then were paraffin-embedded after dehydration through a series of graded ethanol solutions and xylene. Next, the ovaries used for follicle quantification were sectioned serially at 5 µm intervals for H&E staining. Follicles at various stages of development, including primordial, primary, secondary, preantral, and antral follicles, were characterized as described previously^37^. And the number of follicles in each stage was counted in every third section throughout the whole ovary by double-blind method. To avoid repeated counting of the same follicle, only follicles containing an oocyte with visible germinal vesicle nuclei stained by hematoxylin were counted.

### Early embryo collection and RT-qPCR

The method for collecting oocytes was described in the previous section. To prepare sperm from 12- to 16-week-old male mice, the cauda epididymitis was removed from a mature male mouse. Blood and adipose tissue were removed from the surface, and the caudal (enlarged) portion was excised using a pair of fine scissors. The excised specimens were compressed to release a dense mass of spermatozoa from which drops were collected and placed in a dish containing 100 μL of HTF medium and cultured at 37 °C in a CO_2_ incubator for 50–60 min. Five to ten microliters of sperm suspension were collected and introduced into each drop containing COCs. After insemination for 5–6 h, pronuclear formation was verified following fertilization, and prior to allocation for culture, all embryos were washed several times with 50 μL drops of CZB medium. For each biological replicate, oocytes from all female mice were pooled, and sperm from one male mouse was used for fertilization. All media and oil used were preequilibrated overnight at 5% CO_2_ in air at 37 °C. In vitro fertilized 2-cell stage embryos were vitrified 46–48 h after hCG injection (4-cell, 58–60 h; 8-cell, 67–70 h; and blastocyst, 89–93 h).

Collected embryos were lysed to release all RNAs, which then were reverse transcribed into the first cDNA strands by using the Smart-Seq2 method. cDNA was used for further RT-qPCR analysis.

### Sperm sample collection and sRNA sequencing

#### Sperm isolation, sRNA library construction, and sequencing

The mature sperm of male mice were collected from the cauda epididymis as described previously. Briefly, sperm were released from the cauda epididymis into 1 mL of PBS maintained at 37 °C for 15 min, followed using a 40-μm cell strainer to remove tissue debris. The sperm were then treated with somatic cell lysis buffer (0.1% SDS, 0.5% Triton X-100 in DEPC H_2_O) for 40 min on ice and pelleted at 600× *g* for 5 min to remove somatic cell contaminants. After removing the suspension, it was washed twice through 10 mL of PBS and pelleted at 600× *g* for 5 min. The sperm pellet was resuspended, rinsed twice in 10 mL of PBS, and then pelleted at 600× *g* for 5 minutes. The sperm pellet was added to TRIzol Reagent and homogenized, followed by RNA extraction. sRNA libraries were created using the TruSeq Small RNA Sample Pre-Kit (Illumina), and the sRNA libraries were prepared followed by library quality validation for sequencing. Berry Genomics (China) performed the deep sequencing.

#### Quality control and data analysis

FastQC was used to evaluate the sequencing quality, and Cutadapt and Trimmomatic were then used to trim sequence reads that matched any of the following parameters of the standard quality control criteria: (i) reads with N; more than 4 bases whose quality score was lower than 10 or more than 6 bases whose quality score was lower than 13; (ii) reads with 5’ primer contaminants or without 3’ primers; (iii) reads without the insert tag; (iv) reads with poly A; and (v) reads shorter than 18 nt. The clean reads were obtained after data filtration. Precursor and mature miRNA sequences, tRNA sequences, piRNA sequences, and rRNA sequences were obtained from miRBase v21, GtRNAdb, piRBase, and rRNAdb, respectively. Bowtie was used to align clean reads to these reference databases for annotation. To annotate miRNAs, only candidates with one mismatch and no more than two shifts were counted as miRNA matches. To annotate piRNAs, only candidates with one mismatch were counted as piRNA matches. SPORTS 1.1 based on Bowtie was used for tsRNA and rsRNA annotation. DEseq2 was used to perform differential expression detection on tabulated read counts.

The sRNA target prediction databases Diana Tools (http://diana.imis.athena-innovation.gr/DianaTools/index.php) and miRWalk (http://mirwalk.umm.uni-heidelberg.de/) were used to predict the target genes of the differentially expressed sRNAs.

### Single-cell RNA-sequencing (scRNA-seq)

#### RNA library construction and sequencing

Single-cell suspensions (1 × 10^5^ cells/mL) in PBS (HyClone) were loaded into microfluidic devices using the Singleron Matrix® Single Cell Processing System (Singleron). Subsequently, the scRNA-seq libraries were constructed according to the protocol of the GEXSCOPE® Single Cell RNA Library Kit (Singleron). Individual libraries were diluted to 4 nM and pooled for sequencing. Finally, pools were sequenced on an Illumina HiSeq X with 150-bp paired-end reads.

#### Data analysis

Raw reads were processed to generate gene expression profiles using an internal pipeline. Briefly, the cell barcode and UMI were extracted after filtering read one without poly T tails. Adapters and poly A tails were trimmed (fastp V1) before aligning read two to the mouse reference genome (GRCm38, version 38). Reads with the same cell barcode, UMI and gene were grouped together to calculate the number of UMIs of genes in each cell. The UMI count tables of each cellular barcode were employed for further analysis. Cell type identification and clustering analysis were performed by the Seurat program. Furthermore, the Seurat program (http://satijalab.org/seurat/, R package, v.3.0.1) was applied for the analysis of RNA-seq data. UMI count tables were loaded into R using the read.table function.

### Sperm DNA extraction and methylation sequencing

#### DNA library construction and sequencing

Sperm genomic DNA extraction was performed using a phenol-chloroform protocol according to a previous description. Briefly, sperm were seeded into lysis buffer. DNA was released after proteinase treatment at 50 °C and then subjected to bisulfite conversion (EZ DNA Methylation-Gold Kit, ZYMO RESEARCH). After column-based purification, DNA was complemented with the biotinylated random primer Bio-P5-N9 and 50 units of Klenow polymerase (3′ to 5′ exo-, New England BioLabs). This random priming was repeated five times in total. The second strands were synthesized using another random primer, P7-N9, and final libraries were generated after 8 to 12 cycles of PCR amplification with the Illumina universal PCR primer and Illumina indexed primer. Sequencing data were generated on the Illumina HiSeq 4000 platform.

#### Data analysis

fastp was used to evaluate the sequencing quality and trim sequence reads that matched any of the following parameters of the standard quality control criteria: (i) adapter dimer reads; (ii) reads with *N* > 5 were removed; (iii) the proportion of bases with a quality value of < 20 in the sequence exceeds 10% of the total length; (iv) reads shorter than 50 bp. Reads that passed quality control were mapped to the mouse reference genome (mm10) using Bismark with 28 paired-end alignment mode (parameters: --nondirectional --fastq).

#### DMRs

DMRs at CG dinucleotides were identified by performing an analysis of variance (ANOVA)-like test for differential methylation with the DSS v2.44.0 R/Bioconductor package (smoothing = TRUE, smoothing. Span = 200, delta = 0, p.threshold = 1E–05, minlen = 50, minCG = 3, dis.merge = 100, and pct.sig = 0.5).

### Luciferase reporter assay

The reporter vector pmirGLO-LHCGR, pmirGLO-NSD1, pmirGLO-TET1 or the respective mutation vector (Tsingke Biotechnology Co., Ltd.) with piR016061, miR6240, piR033435 or piR006695-binding site mutations was co-transfected with piR016061, miR6240, piR033435 or piR006695 mimic or scrRNA (RiboBio Co., Ltd.) into 293T cells. Firefly and Renilla luciferase activities were measured 48 h after transfection using the Dual-Luciferase Reporter Assay System (Promega, Cat# E1910) according to the manufacturer’s instructions.

### Bisulfite sequencing

Bisulfite-treated DNA PCR products were purified with a Gel Extraction Kit (Vazyme, Nanjing, China) and cloned into the pCE3 Blunt-Zero Vector following amplification. Individual clones were sequenced utilizing conventional Sanger sequencing (Sangon Biotech, Shanghai, China). QUMA was used to analyze the data (http://quma.cdb.riken.jp/). The employed primers are listed in Supplementary Table S7.

### The in vitro transcription

To generate mRNAs for microinjection experiments aimed at rescuing Tet1 function, we first constructed plasmids by cloning the wild-type *Tet1* or a *Tet1* mutant with a YRA coding sequence replacing the *HKD* coding sequence into the pCDNA3.1 vector. Next, we linearized the plasmids using the *ACC65*I restriction endonuclease reaction system (NEB). The resulting purified DNA fragments were used as a template for in vitro transcription with the T7 High Yield RNA Transcription Kit (Vazyme), following the manufacturer’s instructions. We then purified the RNA using VAHTS RNA Clean Beads (Vazyme). Finally, we aliquoted and stored the synthesized RNA at –80 °C until use.

### Supplementary information

Supplementary Information

## Data Availability

The data that support the findings of this study are available from the corresponding author upon reasonable request.
