# Supplementary material for: Inter- and trans-generational impacts of real-world PM2.5 exposure on male-specific primary hypogonadism
Source: Cell Discov. 2024 Apr 23;10:44. doi: 10.1038/s41421-024-00657-0 (PMC11035589; doi:10.1038/s41421-024-00657-0)
Supplement: Supplementary file 1 — Supplementary Information [file 41421_2024_657_MOESM1_ESM.pdf]

Supplemental Information

Inter- and Trans-generational Impacts of Real-world $PM_{2.5}$ Exposure on Male-specific Primary Hypogonadism

Xiaoyu Wei[1,2]†, Zhonghao Zhang[3]†, Yayun Gu[1,2]†, Rong Zhang[4], Jie Huang[1], Feng Li[1], Yuanlin He[1,2], Shuai Lu[1,2], Yifei Wu[1,2], Wentao Zeng[1], Xiaorui Liu[1], Chenzi Liu[1,2], Jinyi Liu[3], Lin Ao[3], Fuquan Shi[3], Qing Chen[3], Yuan Lin[1,2], Jiangbo Du[1,2], Guangfu Jin[1,2], Yankai Xia[1,2], Hongxia Ma[1,2], Yuxin Zheng[5], Ran Huo[1]*, Jia Cao[3]*, Hongbing Shen[1,2]*, Zhibin Hu[1,2]*

This file includes:

Supplementary Figures S1-10

Figure legends

Supplementary Tables S1-7

Supplementary Fig S1

a

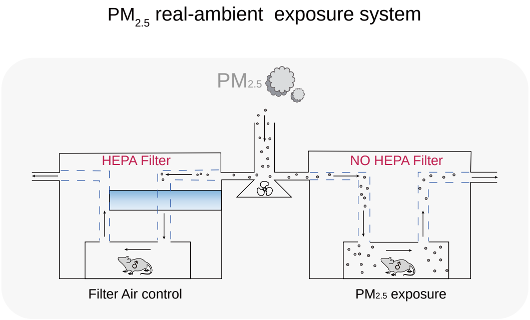

b

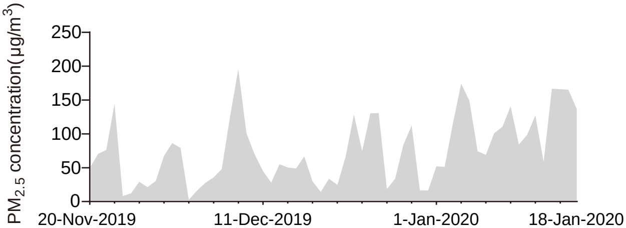

Supplementary Fig S2

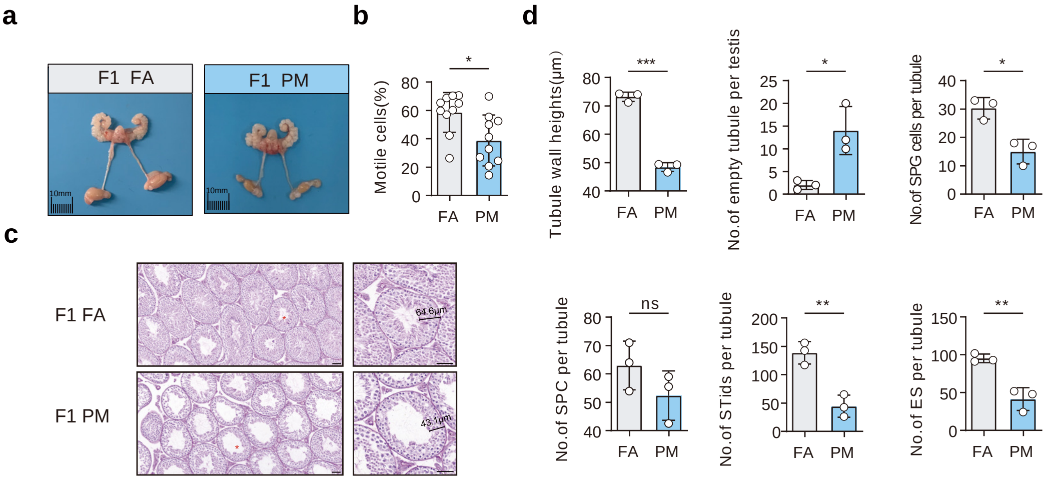

Supplementary Fig S3

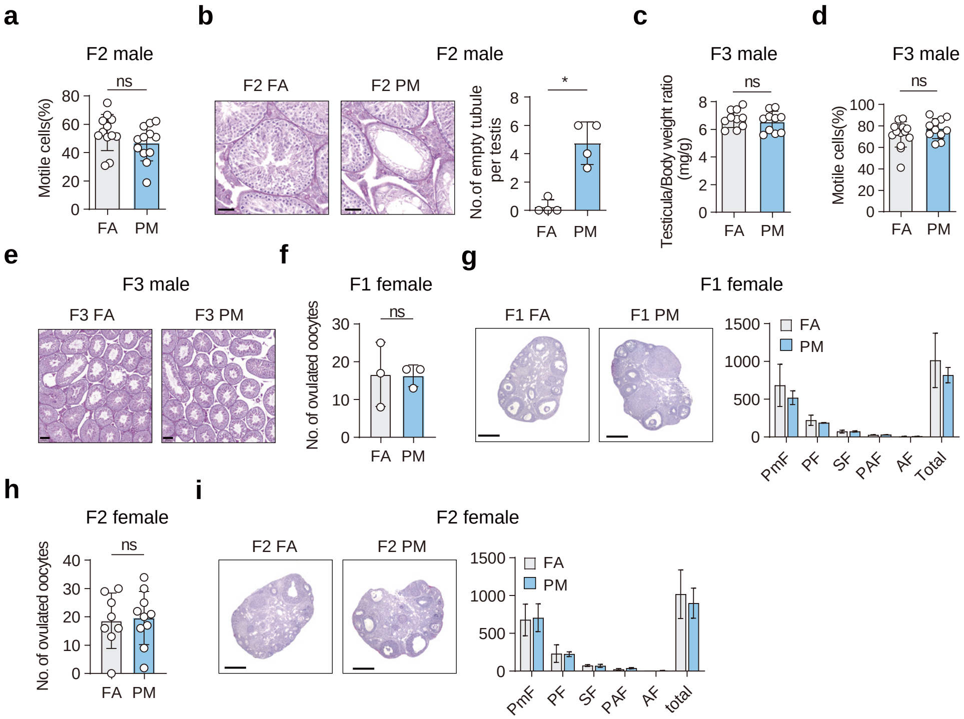

Supplementary Fig S4

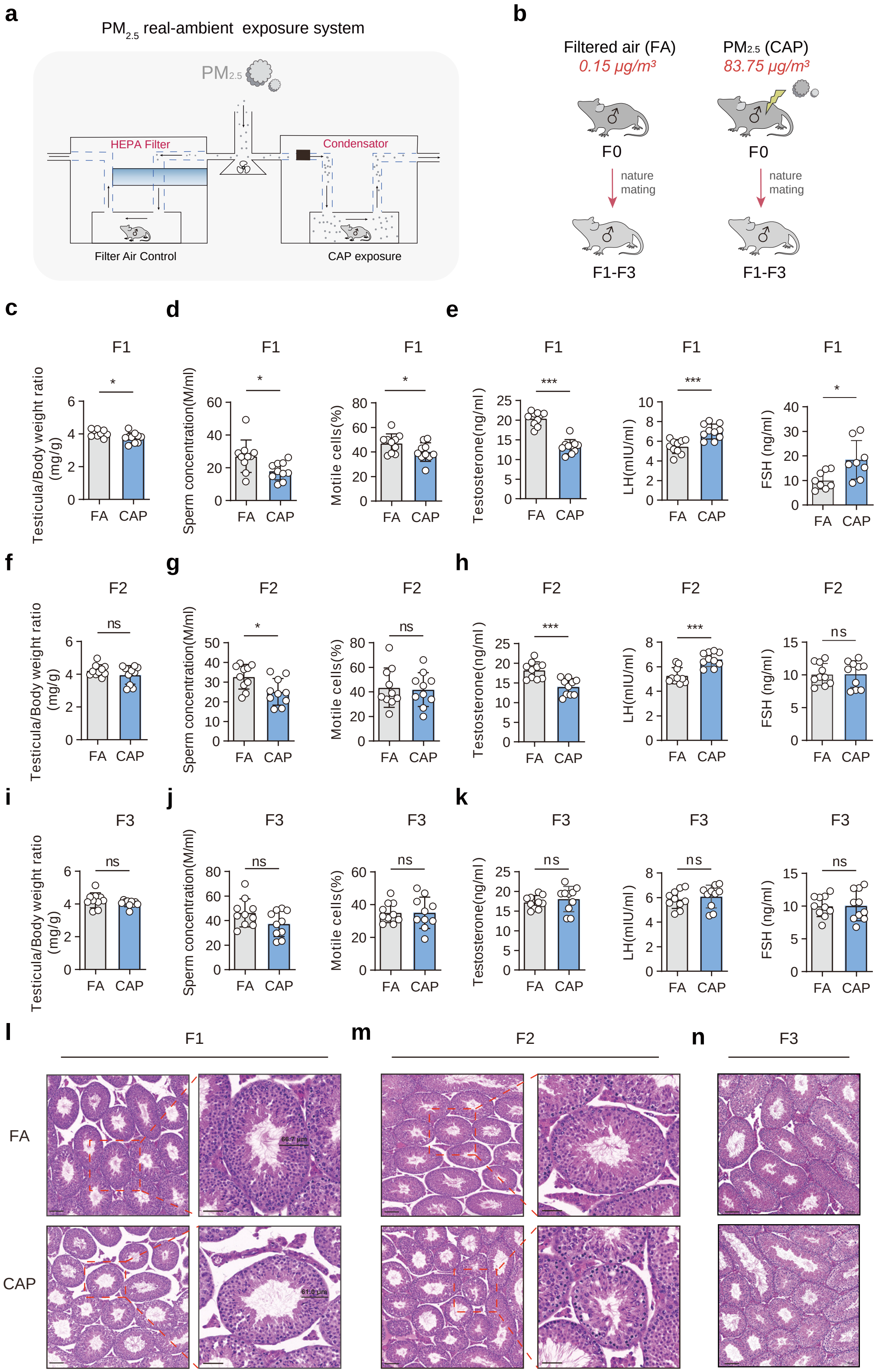

Supplementary Fig S5

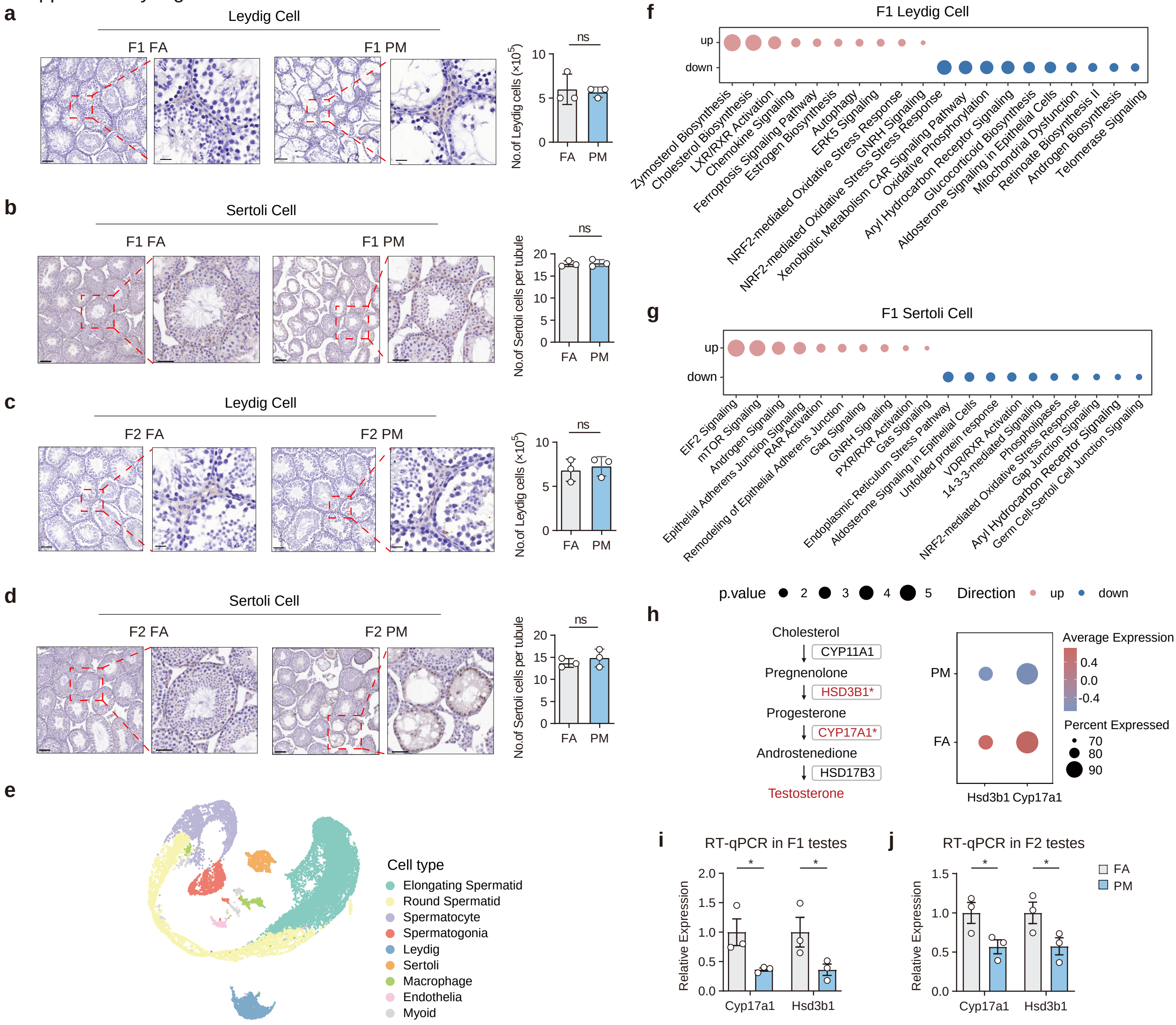

Supplementary Fig S6

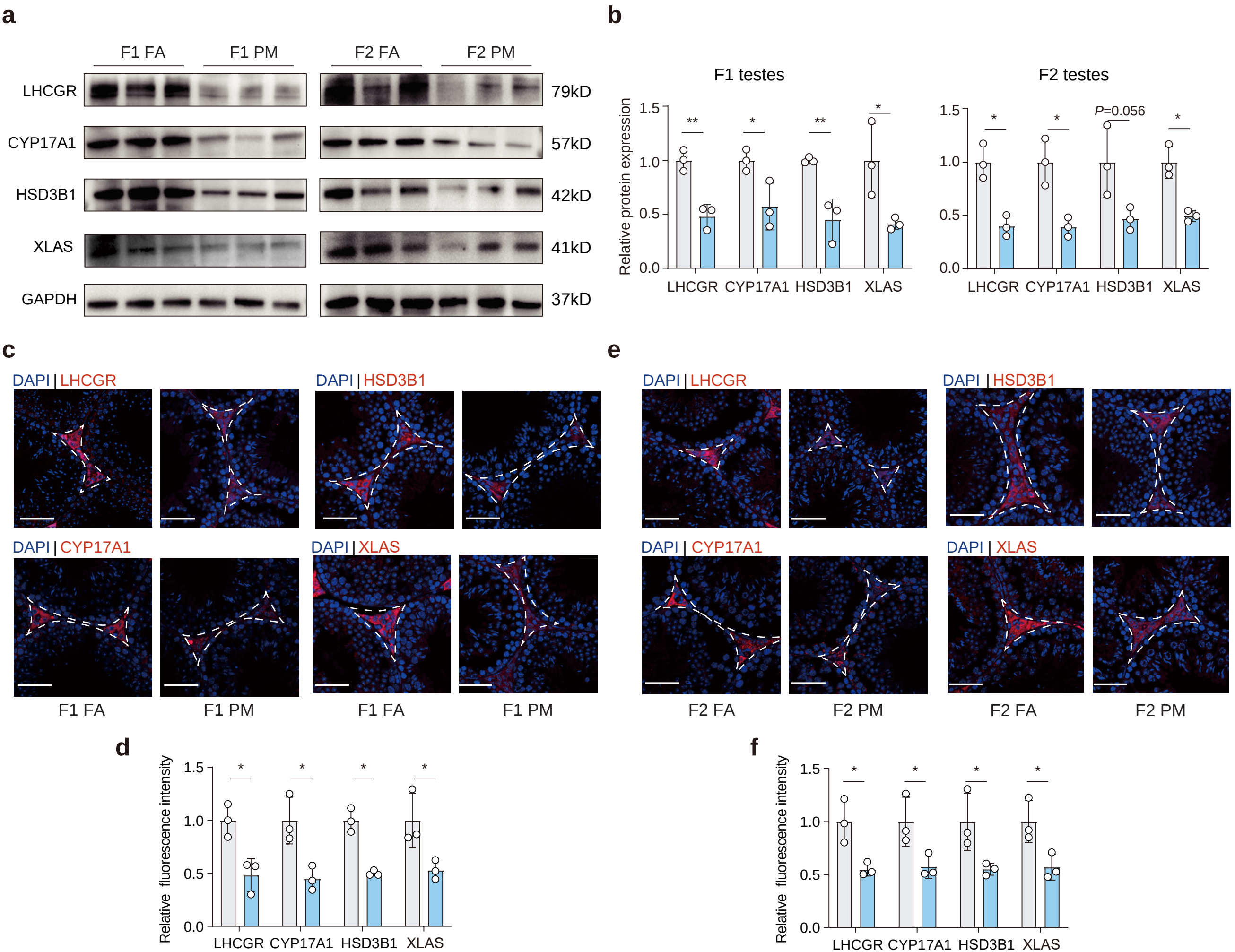

Supplementary Fig S7

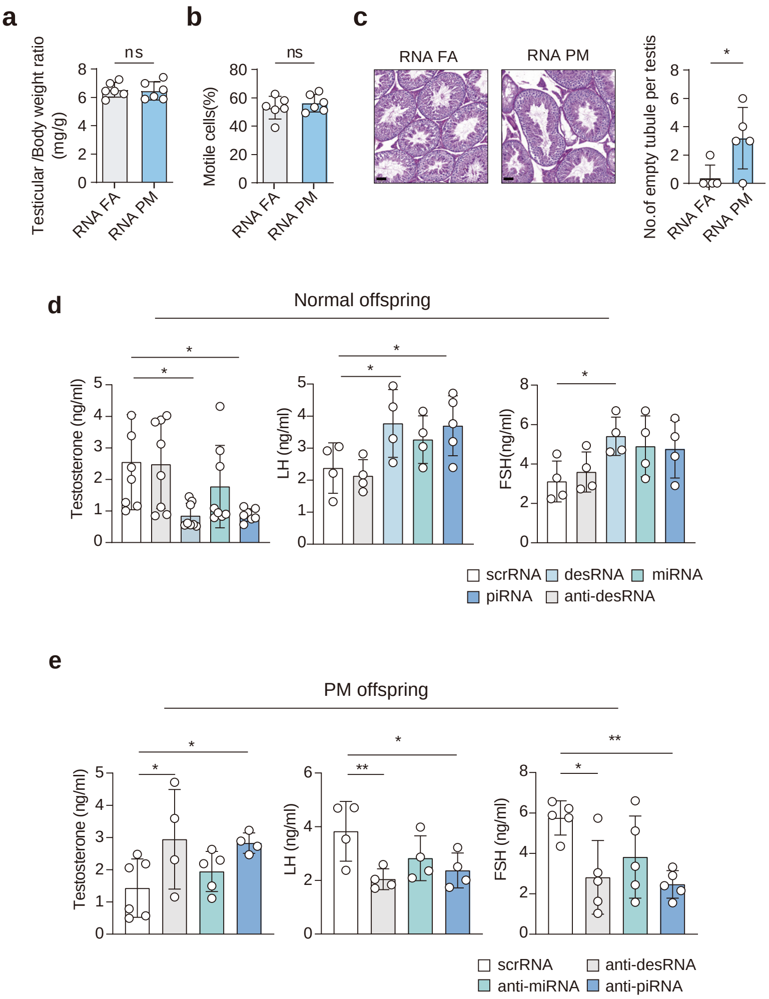

Supplementary Fig S8

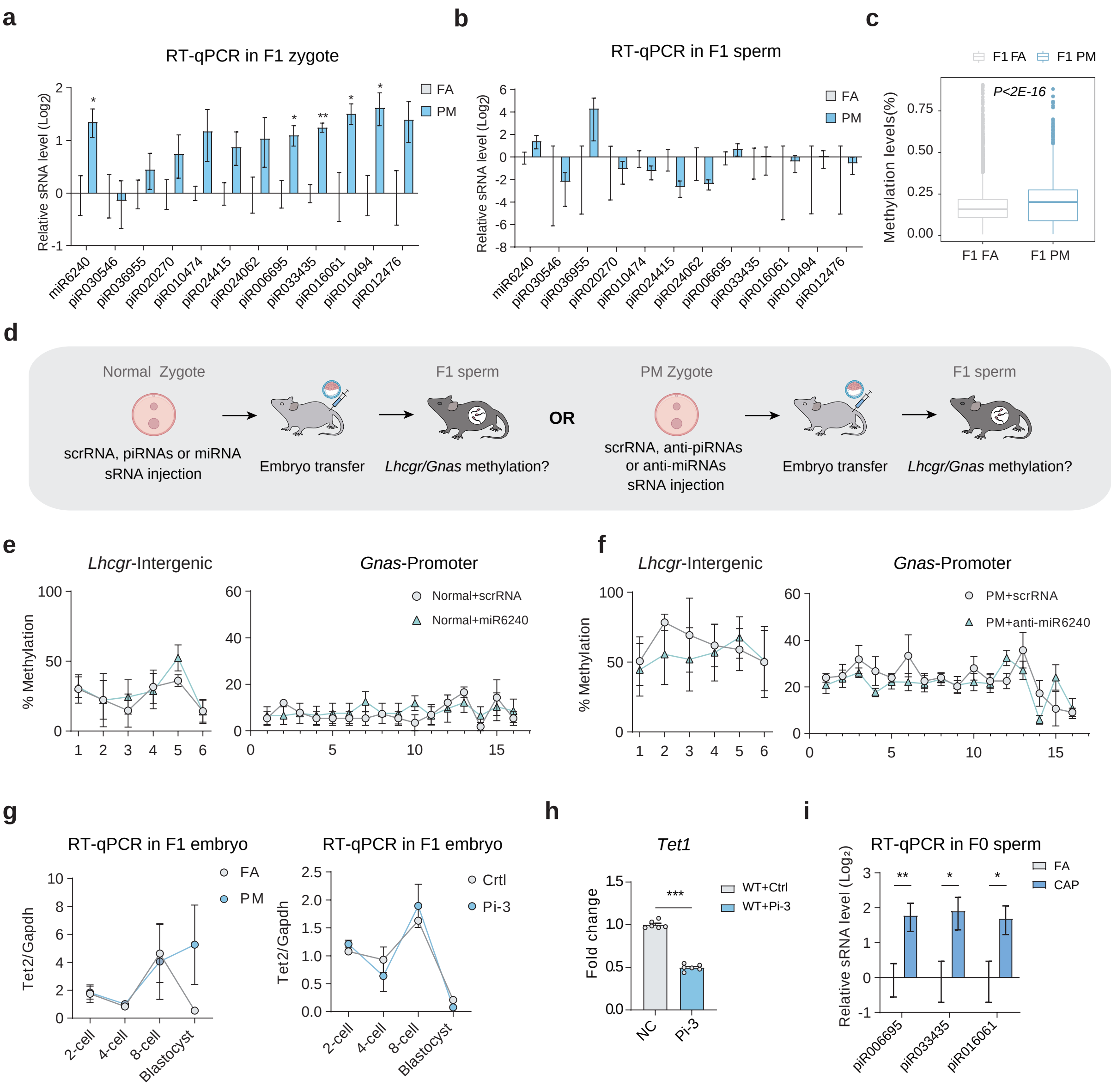

Supplementary Fig S9

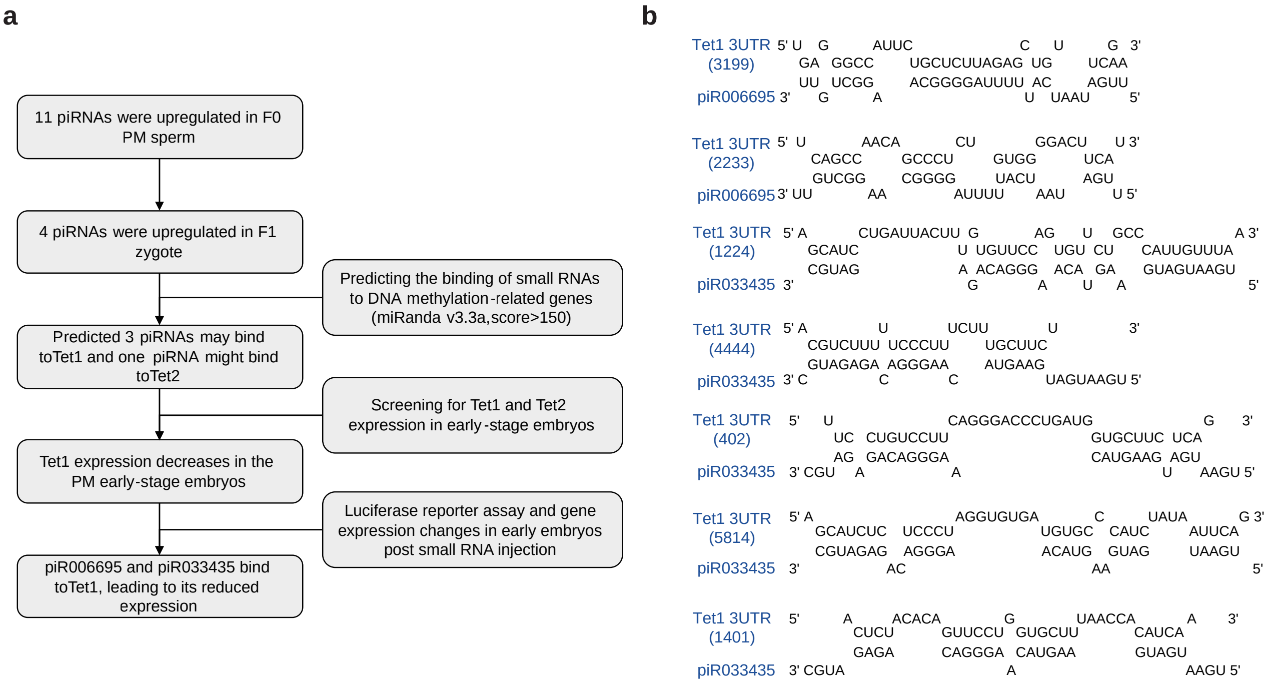

Supplementary Fig S10

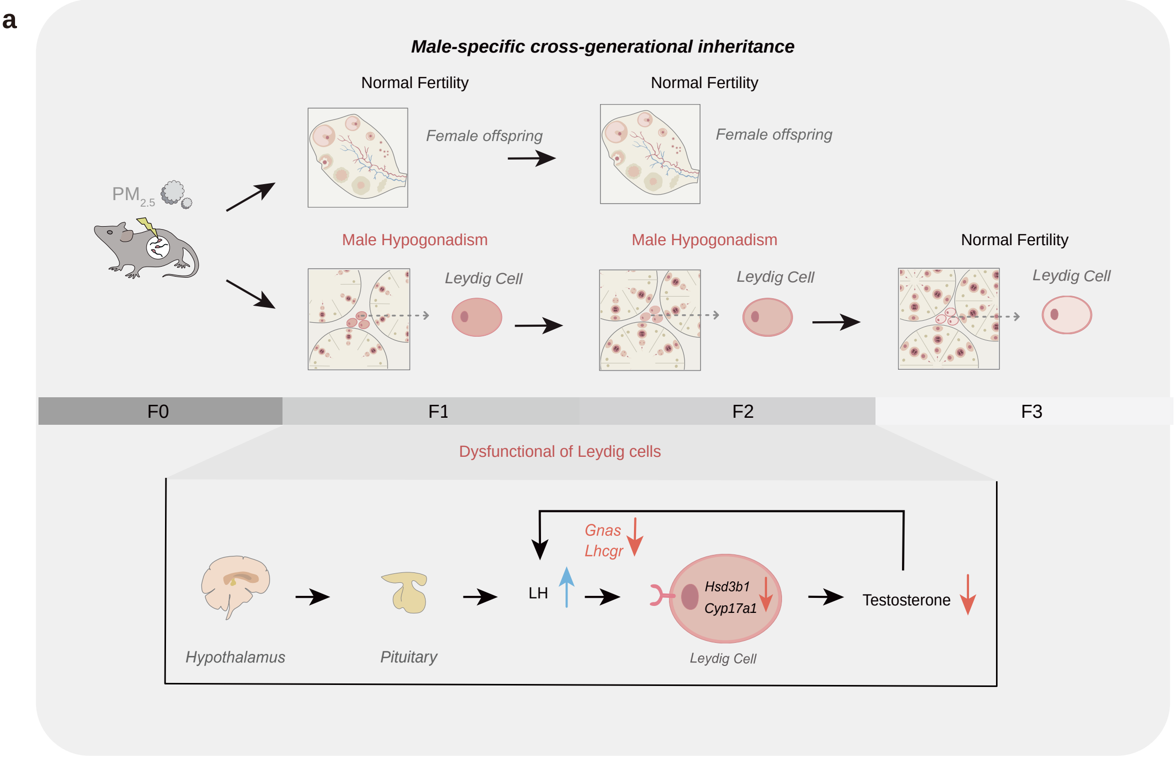

Supplementary Fig. S1. The experimental design and the characteristics and concentration of PM in the exposure chambers and ambient air.

a Diagram of a real-ambient PM exposure system. **b** The mean daily concentration of $PM_{2.5}$ in the PM exposure chamber over the duration of the experiment (60 days).

Supplementary Fig. S2. Paternal $PM_{2.5}$ exposure induces intergenerational transmission of F2 primary male hypogonadism.

a Morphology of the genital tracts in adult F1 males (P160-P180). **b** Sperm motility analyzed by CASA in adult F1 males (P160-P180). n = 10 per group. **c** Left: Periodic acid-Schiff/hematoxylin-stained testicular cross-sections of F1 males (scale bars, 100 μm). Right: Representative histological images of testis sections show tubule wall heights of adult F1 males (P160-P180) at the same stage of spermatogenesis (Stage VII; scale bars, 50 μm). **d** The mean wall heights of seminiferous tubules at Stage VII. The abundances of the indicated germ cells in the Stage VII tubules. Spermatogonia (SPG), meiotic spermatocytes (SCytes), postmeiotic haploid round spermatids (STids), elongating spermatids (ES) per tubule and number of empty tubules per testis. Values are presented as the mean ± SEM. For statistical analysis, *p* values were calculated by t test. Statistical significance for all analyses was $^{*}p < 0.05$; $^{**}p < 0.005$; $^{***}p < 0.0005$.

Supplementary Fig. S3. Paternal $PM_{2.5}$ exposure induces transgenerational transmission of F2 primary male hypogonadism.

a Sperm motility analyzed by CASA in adult F2 males (P160-P180). n = 12 per group. **b** Left: Periodic acid-Schiff/hematoxylin-stained testicular cross-sections of F1 males (P160-P180) (scale bars, 50 μm). Right: Number of empty tubules per testis (P160-P180) (n = 3). **c** Testicular to body weight ratio in adult F3 males (P160-P180). n = 10 per group. **d** Sperm motility analyzed by CASA in adult F3 males (P160-P180) (FA: n = 12, PM: n = 10). **e** Periodic acid-Schiff/hematoxylin-stained testicular cross-sections of F3 males (scale bars, 100 μm). **f** and **h** Number of ovulated oocytes obtained after superovulation of adult F1 (n = 3) and F2 females (FA: n = 8, PM: n = 10). **g** and **i** HE-stained ovarian cross-sections and quantitative analysis of follicles at different stages in ovaries of F1 and F2 females (scale bars, 100 μm). Unilateral ovaries were isolated from mice of two groups and counted in each experiment. n = 3 per group. Values are presented as the mean ± SEM. For statistical analysis, p values were calculated by *t* test. Statistical significance for all analyses was $*p < 0.05$; $**p < 0.005$; $***p < 0.0005$.

Supplementary Fig. S4. An external model verified the transgenerational transmission of $PM_{2.5}$ exposure on male fertility.

a Diagram of a real-ambient CAP exposure system. **b** Schematic illustration of the experimental design. F0: Adult male mice were randomized to the filtered air (FA) and concentrated $PM_{2.5}$ exposure (CAP) groups. The mice of each group were naturally

mated with normal female mice to generate the F1 offspring. F2 offspring were produced by mating F1 males with normal females, and F3 offspring were produced in the same way. None of the F1-F3 offspring were directly exposed to $PM_{2.5}$. **c**, **f**, and **i** Testicular to body weight ratio in adult F1-F3 males (P160-P180). n = 10 per group. **d**, **g**, and **j** Sperm concentration (n = 10) and sperm motility (n = 9) in adult F1-F3 males (P160-P180). **e**, **h**, and **k** Plasma testosterone, LH and FSH concentrations in adult F1-F3 males (P160-P180). n >5 per group. **l-m** Histologically stained testicular cross-sections of F1-F3 males are presented, encompassing both low magnification (Scale bars, 100 μm) and an enlarged region of interest (Scale bars, 50 μm). **n** HE-stained testicular cross-sections of F3 males. Values are presented as the mean ± SEM. For statistical analysis, *p* values were calculated by an unpaired two-tailed Student's *t* test. Statistical significance for all analyses was $*p < 0.05$; $**p < 0.005$.

Supplementary Fig. S5. scRNA-seq revealed that an altered microenvironment compromise PM F1 testes.

a-d The testicular cross-sections of F1-F2 were stained with Leydig cell marker (INSL3) and Sertoli cell marker (SOX9), counterstained with hematoxylin (P160-P180). Left: Optical microscope images depict immunohistochemical staining for Leydig cell marker (brown) and Sertoli cell marker (brown), featuring both low magnification (Scale bars, 100 μm) and an enlarged region of interest (Scale bars, 20 μm). Right: The number of Sertoli cells per seminiferous tubules (P160-P180) and the total number of Leydig cells

per testis (P160-P180). **e** Dimension reduction presentation (via UMAP) of single-cell transcriptome data from F1 testes (n = 17,587). Each dot represents a single cell and is colored according to its cell type identity. Two replicates per group, each replicate captures ~4,000 cells. Using previously described cell-type specific markers, we identified all major germ cell populations covering the full developmental spectrum, including spermatogonia (SPG), meiotic spermatocytes (SCytes), postmeiotic haploid round spermatids (STids), and elongating spermatids (ES), and we identified somatic cells, including Leydig cells, Sertoli cells, macrophages, endothelial cells, and myeloid cells. **f** and **g** IPA terms enriched in differentially expressed genes in Leydig cells and Sertoli cells between FA-F1 and PM-F1. Significance is indicated as the -log10 *p* value. **h** Left: Essential enzymes for testosterone biosynthesis. Red characters mean which gene were downregulated in scRNA-seq. Right: Dot plot showing the gene expression of *Cyp17a1* and *Hsd3b1* in Leydig cells. **i** and **j** RT-qPCR analyses using primers against testosterone synthesis-related genes were performed in F1 and F2 testis tissues. n = 3 per group. For statistical analysis, *p* values were calculated by *t* test. Statistical significance for all analyses was $^*p < 0.05$.

Supplementary Fig. S6. Protein expression in the testicular mesenchyme of F1 and F2.

a and **b** Western Blot analysis and quantification of LHCGR, CYP17A1, HSD3B1 and GNAS (XLAS) from F1 and F2 testes. **c** and **e** Representative immunostaining images of

LHCGR, CYP17A1, HSD3B1, XLAS (in red), and DAPI (in blue) in the testicular mesenchyme of F1 and F2 (P160-P180). Scale bar, 50 μm for original pictures. **d** and **f** Relative fluorescence intensity of LHCGR, CYP17A1, HSD3B1, XLAS in the testicular mesenchyme of F1 and F2. n = 3 per group. For statistical analysis, *p* values were calculated by *t* test. Statistical significance for all analyses was $*p < 0.05$.

Supplementary Fig. S7. Sperm RNA conferred paternal transmission of male hypogonadism.

a and **b** Testicular to body weight ratio (n = 6 per group) and sperm motility (n = 6 per group) of RNA-FA and RNA-PM offspring (P160-P180). **c** Periodic acid-Schiff/hematoxylin-stained testicular cross-sections and number of empty tubules of RNA-FA and RNA-PM offspring (P160-P180) (scale bars, 50 μm). **d** Plasma testosterone, LH and FSH concentrations of Normal male offspring born to zygotes injected with scrRNA, anti-desRNA, miRNA, piRNA and desRNA (P160-P180). **e** Plasma testosterone, LH and FSH concentrations of PM male offspring born to zygotes injected with anti-miRNA, anti-piRNA and anti-desRNA (P160-P180). Values are presented as the mean ± SEM. For statistical analysis, *p* values were calculated by *t* test (**a**-**c**). Kruskal-Wallis one-way ANOVA followed by Dunnett's correction was used (**d** and **e**). Statistical significance for all analyses was $*p < 0.05$.

Supplementary Fig. S8. Increased piRNAs expression in F0 sperm contributes to hypermethylation of *Lhcgr* and *Gnas* in F1 sperm.

a RT-qPCR analysis of the expression of miRNA and piRNAs in the zygotes derived from FA-F1 vs. PM-F1. n = 4 per group. **b** RT-qPCR analysis of the expression of DESs in the sperm derived from FA-F1 vs. PM-F1. n = 3 per group. **c** Whole-genome methylation levels of PM and FA in F1 sperm detected by WGBS. **d** Model illustrating the methylation level of F1 sperm from PM or Normal mice injected with synthesized sRNAs. **e** and **f** Bisulfite sequencing analysis of the *Lhcgr* intergenic region and *Gnas* promoter in scrRNA-Normal, miR6240-Normal, anti-miR6240-PM, and anti-scrRNA-PM (n = 3-4 per group). **g** Left: Line chart of the relative expression levels of *Tet2* in early embryos. Zygotes derived from FA-F1 or PM-F1 were cultured, and the gene transcriptional changes were assessed across early embryonic stages (n = 3). Right: Line chart of the relative expression levels of *Tet1* in early embryos. Zygotes were injected with scrRNA or Pi-3 (piR033435, piR016061, and piR006695 mimics), the gene transcriptional changes were assessed across early embryonic stages (n = 3). **h** Luciferase reporter assay of the *Tet*1 3′UTR reporters in 293T cells 48 h after transfection (n = 6). WT: wild type; Ctrl: scrambled negative control. Pi-3: piR033435, piR016061, and piR006695 mimics. **i** RT-qPCR analysis of the expression of piR033435, piR016061, and piR006695 in the sperm derived from FA-F1 vs. CAP-F1. n =3 per group. Values are presented as the mean ± SEM. For statistical analysis, *p* values were calculated by

unpaired *t* test (**c** and **h**), multiple unpaired *t* test (**a**, **b**, and **i**), two-way ANOVA followed by Dunnett's multiple comparisons test (**e**, **f**, and **g**). Statistical significance for all analyses was $*p < 0.05$; $***p < 0.0005$.

Supplementary Fig. S9. The process of selecting potential-effect piRNAs and predicting piRNA targets.

a and **b** The selection process of piRNAs for potential effects and the prediction of target genes for effective piRNAs.

Supplementary Fig. S10. Model illustrating the transgenerational effect of $PM_{2.5}$ exposure on male fertility.

a Paternal exposure to $PM_{2.5}$ can induce, in a sex-specific manner, transgenerational effects on male offspring, and F1 and F2 male offspring showing male primary hypogonadism traits and display impairment of spermatogenesis and subfertility. However, female offspring present with normal fertility. The main defect of testes is the dysfunctional of Leydig cells. *Lhcgr* and *XLas,* as well as receptor-stimulated cAMP production, are decreased in Leydig cells, leading to reduced expression of *Cyp17a1* and *Hsd3b1*, thereby reducing testosterone and enhancing the hypothalamic-pituitary-gonadal (HPG) axis. Future consideration should be given to the development of prevention and treatment methods, including prenatal consultation, for those likely to be exposed to $PM_{2.5}$.

Supplementary Table S1-S7

Supplementary Table S1. Significantly changed sRNA between PM and FA sperm.

$p_{adj} < 0.01$.

ID	FA	PM	log2FoldChange	*p*-value	p_{adj}
miR6240	14.6	136.97	3.24	4.49E-08	3.25E-05
piR010474	25.3	104.56	2.04	2.33E-06	9.79E-04
piR012476	33.01	130.58	1.98	2.51E-07	1.76E-04
piR016061	43.59	152.43	1.81	1.62E-08	3.40E-05
piR036955	25.13	87.38	1.8	8.33E-06	2.06E-03
piR020270	59.34	204.5	1.78	9.27E-09	3.40E-05
piR024062	388.5	1,294.14	1.74	3.13E-08	3.80E-05
piR030546	35.51	108.56	1.61	6.38E-05	7.07E-03
piR010494	40.56	122.21	1.6	1.68E-05	3.22E-03
piR024415	33.99	102.58	1.59	1.44E-05	3.02E-03
piR033435	48.21	141.51	1.55	8.91E-06	2.08E-03
mature-tRNA-Glu-CTC	220,700.66	642,202.72	1.54	1.35E-03	9.89E-03
piR006695	42.07	119.57	1.51	2.63E-05	4.43E-03
piR011982	6465.54	2,186.93	-1.56	7.56E-05	7.58E-03
piR034411	128.46	29.4	-2.15	8.17E-07	4.91E-04
piR027735	25.09	105.04	2.06	3.61E-08	3.80E-05
piR011071	27.49	113.81	2.04	5.92E-06	1.92E-03

mature-tRNA-Lys-CTT	10,710.26	40,774.95	1.93	7.16E-04	6.53E-03
mature-tRNA-Lys-TTT	5,266.11	19,364.15	1.88	9.89E-05	1.81E-03
piR019973	73.66	239.74	1.7	5.95E-05	6.92E-03
piR037484	36.02	107.55	1.59	3.38E-05	4.91E-03
piR038289	134.05	47.8	-1.5	5.07E-05	6.41E-03
piR000257	103.69	28.3	-1.9	4.64E-06	1.63E-03

Note: pink lines indicate been verified in RT-qPCR and for further analysis.

Supplementary Table S2. Small RNA target genes.

**Small RNA**	**Gene symbol**	**Small RNA**	**Gene symbol**
piR016061	*Usp32*	piR016061	*Gsdmc4*
piR006695	*Kdm7a*	piR006695	*Zfp709*
piR033435	*Cabp5*	piR006695	*Ldhal6b*
piR033435	*Slc4a1*	piR016061	*Kcnma1*
piR033435	*Ankrd17*	piR010494	*Actl6a*
piR033435	*Prl5a1*	piR033435	*Nlgn1*
piR016061	*Vmp1*	piR016061	*Nfkb1*
piR010494	*Stk4*	piR016061	*Fbxw22*
piR033435	*Kpna3*	piR016061	*Egf*
piR016061	*Cyp2d22*	piR006695	*Ankrd13c*
piR016061	*Cmtr1*	piR010494	*Creb5*

piR016061	*Lhcgr*	piR033435	*Smim20*
piR016061	*Vps52*	piR033435	*Aldh1l1*
piR016061	*Pde6a*	piR016061	*Wbp1*
piR016061	*Maoa*	piR010494	*Cbl*
piR016061	*Podxl*	piR016061	*Tnrc6a*
piR033435	*Asb1*	piR006695	*Picalm*
piR016061	*Fbn1*	piR006695	*Zfp654*
piR016061	*Plrg1*	piR006695	*Tnfrsf26*
piR016061	*Gbp3*	piR016061	*Olfr1284*
piR010494	*Pclo*	miR6240	*Osbpl7*
piR016061	*Wasl*	miR6240	*Agfg1*
piR016061	*Ccdc184*	miR6240	*Luc7l2*
piR006695	*Gkn2*	miR6240	*Col26a1*
piR010494	*Slc6a6*	miR6240	*Usp48*
piR016061	*Thap12*	miR6240	*Gabra3*
piR016061	*Cdk16*	miR6240	*Cd59a*
piR033435	*Rab39b*	miR6240	*Elovl3*
piR006695	*Rab39b*	miR6240	*Clk2*
piR016061	*Gpr165*	miR6240	*Crx*
piR010494	*Rwdd4a*	miR6240	*Ezh1*
piR006695	*Phip*	miR6240	*Fut1*
piR016061	*Pank1*	miR6240	*Kcnj3*
piR016061	*Tmed8*	miR6240	*Nsd1*

piR010494	*Lpp*	miR6240	*Trpc5*
piR016061	*Lpp*	miR6240	*Bdkrb2*
piR010494	*Slfn9*	miR6240	*Cdh4*
piR033435	*Kcna2*	miR6240	*Cdk6*
piR016061	*Ap5z1*	miR6240	*Mog*
piR016061	*Vat1*	miR6240	*Stau1*
piR016061	*Ppp2r2d*	miR6240	*Sf1*
piR016061	*Trpa1*	miR6240	*Mta2*
piR006695	*Dmxl1*	miR6240	*Adra1a*
piR016061	*Tmem30b*	miR6240	*Med1*
piR016061	*Hs2st1*	miR6240	*Eloa*
piR006695	*Usp37*	miR6240	*Icosl*
piR006695	*Melk*	miR6240	*Caprin1*
piR033435	*Fndc3b*	miR6240	*Cbln1*
piR006695	*Gm4847*	miR6240	*Prelid3b*
piR016061	*Agps*	miR6240	*Tmem151a*
piR016061	*Thoc6*	miR6240	*Lrrc14b*
piR006695	*4921524J17Rik*	miR6240	*Tlcd5*
piR033435	*Wapl*	miR6240	*Ldc1*
piR033435	*Kctd4*	miR6240	*Map6*
piR006695	*Defb1*	miR6240	*Cluh*
piR006695	*Pura*	miR6240	*Phf20l1*
piR016061	*Fbxo28*	miR6240	*Zcchc2*

piR033435	*Gskip*	miR6240	*Specc1l*
piR033435	*Macir*	miR6240	*Phc3*
piR010494	*Macir*	miR6240	*Bcas3*
piR006695	*Macir*	miR6240	*Rbm41*
piR016061	*Ddx17*	miR6240	*Zmym5*
piR033435	*Pcdhb18*	miR6240	*Arrb2*
piR006695	*Spesp1*	miR6240	*Grip1*
piR016061	*Trim3*	miR6240	*Rtl5*
piR006695	*Taok1*	miR6240	*Cnot6*
piR010494	*Eny2*	miR6240	*Cpeb3*
piR006695	*Phxr2*	miR6240	*Tspan33*
piR010494	*Sorl1*	miR6240	*Ctag2*
piR010494	*Tent5c*	miR6240	*Dcp2*
piR033435	*Smug1*	miR6240	*Mfsd8*
piR010494	*Rap1b*	miR6240	*Rab11fip1*
piR006695	*Zfp608*	miR6240	*Ptprb*
piR016061	*Trp63*	miR6240	*Zfp287*
piR016061	*Adamts6*	miR6240	*Slc38a7*
piR006695	*Togaram1*	miR6240	*Tacc1*
piR033435	*Adam10*	miR6240	*Lypd6*
piR016061	*Rab3gap2*	miR6240	*Ubn2*
piR006695	*B4galt6*	miR6240	*E2f2*
piR010494	*Kcnh7*	miR6240	*Dixdc1*

piR016061	*Abcb10*	miR6240	*Orai2*
piR006695	*Tnfrsf22*	miR6240	*Ntrk3*
piR016061	*Cfl2*	miR6240	*Usp24*
piR016061	*Rasal2*	miR6240	*Igsf3*
piR016061	*Rad51b*	miR6240	*Cacna1c*
piR033435	*Cul2*	miR6240	*Mllt6*
piR033435	*Crem*	miR6240	*Ogt*
piR033435	*Zfp820*	miR6240	*Bach2*
piR016061	*C9orf72*	miR6240	*Dlk1*
piR016061	*Uri1*	miR6240	*Flrt3*
piR016061	*Ncoa1*	miR6240	*Mcf2*
piR006695	*Tmem87a*	miR6240	*Pum2*
piR006695	*Mettl14*	miR6240	*Mkrn1*
piR006695	*Cdc14a*	miR6240	*Tgfbr1*
piR016061	*Arnt*	miR6240	*Pkp1*
piR010494	*Mfn1*	miR6240	*Ylpm1*
piR033435	*A630073D07Rik*	miR6240	*Stx8*
piR010494	*Col11a1*	miR6240	*Dpysl5*
piR016061	*Grid2*	miR6240	*Fndc3b*
piR016061	*Slf2*	miR6240	*Dclk1*
piR016061	*Arid2*	miR6240	*Rlim*
piR016061	*Tmco1*	miR6240	*Ebf2*
piR016061	*Zfp462*	miR6240	*Fam168a*

piR016061	*A230072I06Rik*	miR6240	*Gosr2*
piR006695	*Ttn*	miR6240	*Cbx5*
piR016061	*Ttn*	miR6240	*Dnajc1*
piR016061	*Ttc30b*	miR6240	*Gabrb1*
piR016061	*Zfp449*	miR6240	*Gtf2h1*
piR033435	*Cngb3*	miR6240	*Kif3c*
piR010494	*Foxd1*	miR6240	*Mobp*
piR016061	*Nr2c1*	miR6240	*Ntrk2*
piR010494	*Foxk2*	miR6240	*Cxcl5*
piR010494	*Plaa*	miR6240	*Slc6a2*
piR016061	*Nfib*	miR6240	*Serpinb9*
piR010494	*Crk*	miR6240	*Stk10*
piR010494	*Scn8a*	miR6240	*Terf2*
piR033435	*Slc24a3*	miR6240	*Tgoln1*
piR010494	*Ubr5*	miR6240	*Dpysl3*
piR010494	*Alg11*	miR6240	*Pakap*
piR016061	*Fam90a1a*	miR6240	*Btrc*
piR016061	*Arhgef7*	miR6240	*Creb1*
piR016061	*Cdc42bpa*	miR6240	*Dad1*
piR016061	*Pramel6*	miR6240	*Elavl1*
piR016061	*Ctnnd1*	miR6240	*Itsn1*
piR033435	*Osbpl6*	miR6240	*Pcdh10*
piR016061	*Mospd2*	miR6240	*Pdcd4*

piR006695	*Crim1*	miR6240	*Prkacb*
piR010494	*Dpp10*	miR6240	*Prkg1*
piR006695	*Nr6a1*	miR6240	*Eif2ak2*
piR016061	*Rc3h2*	miR6240	*Sel1l*
piR016061	*Paqr3*	miR6240	*Six6*
piR016061	*Nrk*	miR6240	*Tle4*
piR033435	*Hdx*	miR6240	*Ar*
piR033435	*Cadm2*	miR6240	*Lifr*
piR006695	*Cadm2*	miR6240	*Sp1*
piR016061	*Zbtb20*	miR6240	*Nr4a3*
piR033435	*Pias2*	miR6240	*Gria3*
piR016061	*Tcf4*	miR6240	*Arih1*
piR016061	*Ei24*	miR6240	*Jmy*
piR033435	*Opcml*	miR6240	*Piwil1*
piR006695	*Opcml*	miR6240	*Gpr35*
piR016061	*Arid1b*	miR6240	*Rd3*
piR016061	*Cyth3*	miR6240	*Taf9*
piR033435	*Mrgprf*	miR6240	*Diras2*
piR010494	*Tent4b*	miR6240	*Btbd3*
piR016061	*Dus2*	miR6240	*Zfp597*
piR016061	*Fgf13*	miR6240	*Pgr15l*
piR016061	*Prr14l*	miR6240	*Wdr37*
piR016061	*Nap1l2*	miR6240	*Fgfr1*

piR016061	*Slc31a1*	miR6240	*Mcur1*
piR016061	*Ctsh*	miR6240	*Zfp174*
piR016061	*Fntb*	miR6240	*Ino80d*
piR010494	*Enho*	miR6240	*Arfgef2*
piR016061	*Stau2*	miR6240	*Epb41l5*
piR006695	*Ckmt1*	miR6240	*Mapre2*
piR016061	*Carm1*	miR6240	*Nhsl2*
piR016061	*Gm28043*	miR6240	*Wars*
piR010494	*Nfat5*	miR6240	*Iglon5*
piR016061	*Dpf2*	miR6240	*Nsun6*
piR016061	*Gm20716*	miR6240	*D16Ertd472e*
piR010494	*Cep350*	miR6240	*Rapgef6*
piR010494	*Abi1*	miR6240	*Mospd2*
piR033435	*Sntg1*	miR6240	*Csrnp3*
piR016061	*Iffo1*	miR6240	*Kif1b*
piR006695	*Anapc4*	miR6240	*Ssh2*
piR033435	*Lipo1*	miR6240	*Cnppd1*
piR006695	*Capn7*	miR6240	*Selenoi*
piR016061	*Reps1*	miR6240	*Trub1*
piR010494	*Lonp2*	miR6240	*Ccnt2*
piR006695	*Cask*	miR6240	*Cdyl2*
piR010494	*Hmga2*	miR6240	*Rap2a*
piR033435	*Fgd4*	miR6240	*Zxdc*

piR016061	*Shox2*	miR6240	*Lztfl1*
piR033435	*Wdr26*	miR6240	*Pag1*
piR033435	*Pum2*	miR6240	*Dcp1a*
piR006695	*Pum2*	miR6240	*Dnajc28*
piR016061	*Pum2*	miR6240	*Tab2*
piR016061	*Agtpbp1*	miR6240	*Braf*
piR006695	*Zc3h12c*	miR6240	*Taok1*
piR016061	*Slitrk2*	miR6240	*Marchf2*
piR033435	*Cd200r3*	miR6240	*Mark1*
piR033435	*Ablim3*	miR6240	*Arhgef6*
piR016061	*Ccr2*	miR6240	*9530068E07Rik*
piR010494	*Ms4a1*	miR6240	*Creg2*
piR010494	*Tor1aip1*	miR6240	*Slc24a2*
piR010494	*Rbm26*	miR6240	*Mier3*
piR006695	*Mecom*	miR6240	*Pcgf3*
piR016061	*Slit2*	miR6240	*Zfp462*
piR006695	*Grm7*	miR6240	*Rab8b*
piR033435	*Dip2c*	miR6240	*Atg4b*
piR033435	*Zfhx4*	miR6240	*Fam78a*
piR006695	*Zfhx4*	miR6240	*Taf5*
piR016061	*Mta3*	miR6240	*Lrrc8a*
piR006695	*Gm1553*	miR6240	*Vps26b*
piR016061	*Atp13a4*	miR6240	*Tnfrsf21*

piR033435	*Rxfp1*	miR6240	*N4bp2l2*
piR016061	*Kat6b*	miR6240	*Crem*
piR033435	*Fam83b*	miR6240	*Ubp1*
piR016061	*Resp18*	miR6240	*Arpp21*
piR033435	*1700029H14Rik*	miR6240	*Drp2*

Supplementary Table S3. Small RNA sequences are listed.

ID	Sequence
miR6240	CCAAAGCATCGCGAAGGCCCACGGCG
piR010474	TGATTAGATGAATATGGTGATGTGGCTGAGA
piR012476	TGATACGGGAATCCTAGTGTGCTGGGTAGT
piR016061	TGACAGGACATTGCTGATGGGAACCAGAGT
piR036955	TACAGATGAGAAAATTGAAGGCCAGAAAAGT
piR020270	TAGACTTTGTCCACTGCTCAGTCTCTGGGGT
piR024062	TTGATGGTAATTGGAAGAGCTTCTGGGTGA
piR030546	TGGGATTATAGGCATATACAACTCGTGGCA
piR010494	TGAGAATCGCTACCTTCCCACTGAATGT
piR024415	TAAGAAATCAGTCTTTAGGCATTACCCTCT
piR033435	TGAATGATGAAGTACAAGGGACAGAGATGC
mature-tRNA-Glu-CTC	CCCTGGTGGTCTAGTGGTTAGGATTCGGCG
piR006695	TTGATAATCATTTTTAGGGGCAAGGCTGTT

Supplementary Table S4. Anti-sense of DESs are listed.

Small RNA	Anti-sense
miR6240	CGCCGTGGGCCTTCGCGATGCTTTGG
piR010474	TCTCAGCCACATCACCATATTCATCTAATCA
piR012476	ACTACCCAGCACACTAGGATTCCCGTATCA
piR016061	ACTCTGGTTCCCATCAGCAATGTCCTGTCA
piR036955	ACTTTTCTGGCCTTCAATTTTCTCATCTGTA
piR020270	ACCCCAGAGACTGAGCAGTGGACAAAGTCTA
piR024062	TCACCCAGAAGCTCTTCCAATTACCATCAA
piR030546	TGCCACGAGTTGTATATGCCTATAATCCCA
piR010494	ACATTCAGTGGGAAGGTAGCGATTCTCA
piR024415	AGAGGGTAATGCCTAAAGACTGATTTCTTA
piR033435	GCATCTCTGTCCCTTGTACTTCATCATTCA
mature-tRNA-Glu-CTC	CGCCGAATCCTAACCACTAGACCACCAGGG
piR006695	AACAGCCTTGCCCCTAAAAATGATTATCAA

Supplementary Table S5. qPCR primers are listed.

Mouse	F/R	Sequence (5'-3')
Lhcgr	F	CGCCCGACTATCTCTCACCTA
	R	GACAGATTGAGGAGGTTGTCAAA
Cyp17a1	F	GCCCAAGTCAAAGACACCTAAT
	R	GTACCCAGGCGAAGAGAATAGA
Hsd3b1	F	TGGACAAAGTATTCCGACCAGA
	R	GGCACACTTGCTTGAACACAG

Nsd1	F	ATTTGGGCAAAATTCAAGAGACG
	R	GCCTCCTATTGGCAACTTTCATT
Gnas	F	CAGAGCCTCCATTGGGGTC
	R	GCTTCTCGCTCAACTGGGG
Mature-Trna-Lys-TTT	F	ACACTCCAGCTGGGCCCGGATAGCTCAGTC
Mature-tRNA-Lys-CTT	F	ACACTCCAGCTGGGCCCGGCTAGCTCAGTC
Mature-tRNA-Glu-CTC	F	ACACTCCAGCTGGGCCCTGGTGGTCTAGTG
miR6240	F	ACACTCCAGCTGGGCCAAAGCATCGCGAAG
piR020270	F	ACACTCCAGCTGGGTAGTATGGTGATATTT
piR016061	F	ACACTCCAGCTGGGTGACAGGACATTGCTG
piR024062	F	ACACTCCAGCTGGGTTGATGGTAATTGGAA
piR027735	F	ACACTCCAGCTGGGTAAGAAGGTGTCTAAG
piR012476	F	ACACTCCAGCTGGGTGATACGGGAATCCTA
piR034411	F	ACACTCCAGCTGGGTAAGTGTGAGCATGTA
piR010474	F	ACACTCCAGCTGGGTGATTAGATGAATATG
piR000257	F	ACACTCCAGCTGGGAGCAAAGACAAACTAT
piR011071	F	ACACTCCAGCTGGGTGGATCTTAAATGTCC
piR036955	F	ACACTCCAGCTGGGTACAGATGAGAAAATT
piR033435	F	ACACTCCAGCTGGGTGAATGATGAAGTACA
piR024415	F	ACACTCCAGCTGGGTAAGAAATCAGTCTTT
piR010494	F	ACACTCCAGCTGGGTGAGAATCGCTACCTT
piR006695	F	ACACTCCAGCTGGGTTGATAATCATTTTTA
piR037484	F	ACACTCCAGCTGGGTCTAAAGTCTCTTATC

piR038289	F	ACACTCCAGCTGGGTAAAACAAAGAAGGGA
piR019973	F	ACACTCCAGCTGGGTACAAGAATTGCTGAA
piR030546	F	ACACTCCAGCTGGGTGGGATTATAGGCATA
piR011982	F	ACACTCCAGCTGGGAAAGCAGAGGTAGATG
piR011546	F	ACACTCCAGCTGGGTCAAAGATCGTAATTC
piR034406	F	ACACTCCAGCTGGGTTGTCCATTGAGTGTC
piR012524	F	ACACTCCAGCTGGGGAAAGACATCATGCTG
piR028356	F	ACACTCCAGCTGGGAACTGTGTCTTTTCTG
piR022629	F	ACACTCCAGCTGGGGAAAGACACCAAGCTG
U6	F	ACACTCCAGCTGGGCGCAAATTCGTGAAGC
Reverse	R	CTCAACTGGTGTCGTGGAGTCGG

Supplementary Table S6. Reverse transcription primers for sRNAs are listed.

**sRNA**	RT	**Sequence (5'-3')**
mature-tRNA-Lys-TTT	RT	CTCAACTGGTGTCGTGGAGTCGGCAATTCAGTTGAGAGTCTGATGC
mature-tRNA-Lys-CTT	RT	CTCAACTGGTGTCGTGGAGTCGGCAATTCAGTTGAGAGTCTCATGC
mature-tRNA-Glu-CTC	RT	CTCAACTGGTGTCGTGGAGTCGGCAATTCAGTTGAGCGCCGAATCC
miR6240	RT	CTCAACTGGTGTCGTGGAGTCGGCAATTCAGTTGAGCGCCGTGGGC

piR020270	RT	CTCAACTGGTGTCGTGGAGTCGGCAATTCAGTTGAGA CCTGTAGTC
piR016061	RT	CTCAACTGGTGTCGTGGAGTCGGCAATTCAGTTGAGA CTCTGGTTC
piR024062	RT	CTCAACTGGTGTCGTGGAGTCGGCAATTCAGTTGAGT CACCCAGAA
piR027735	RT	CTCAACTGGTGTCGTGGAGTCGGCAATTCAGTTGAGG AACCACATA
piR012476	RT	CTCAACTGGTGTCGTGGAGTCGGCAATTCAGTTGAGA CTACCCAGC
piR034411	RT	CTCAACTGGTGTCGTGGAGTCGGCAATTCAGTTGAGA CATATACAT
piR010474	RT	CTCAACTGGTGTCGTGGAGTCGGCAATTCAGTTGAGT CTCAGCCAC
piR000257	RT	CTCAACTGGTGTCGTGGAGTCGGCAATTCAGTTGAGG CAGGAATGA
piR011071	RT	CTCAACTGGTGTCGTGGAGTCGGCAATTCAGTTGAGA CATGTGACA
piR036955	RT	CTCAACTGGTGTCGTGGAGTCGGCAATTCAGTTGAGA CTTTTCTGG
piR033435	RT	CTCAACTGGTGTCGTGGAGTCGGCAATTCAGTTGAGG CATCTCTGT

piR024415	RT	CTCAACTGGTGTCGTGGAGTCGGCAATTCAGTTGAGA GAGGGTAAT
piR010494	RT	CTCAACTGGTGTCGTGGAGTCGGCAATTCAGTTGAGA CATTCAGTG
piR006695	RT	CTCAACTGGTGTCGTGGAGTCGGCAATTCAGTTGAGA ACAGCCTTG
piR037484	RT	CTCAACTGGTGTCGTGGAGTCGGCAATTCAGTTGAGG AAATGCACC
piR038289	RT	CTCAACTGGTGTCGTGGAGTCGGCAATTCAGTTGAGG GCAGGGAAT
piR019973	RT	CTCAACTGGTGTCGTGGAGTCGGCAATTCAGTTGAGA CCCTCAGAC
piR030546	RT	CTCAACTGGTGTCGTGGAGTCGGCAATTCAGTTGAGT GCCACGAGT
piR011982	RT	CTCAACTGGTGTCGTGGAGTCGGCAATTCAGTTGAGG AAGTCCATC
piR011546	RT	CTCAACTGGTGTCGTGGAGTCGGCAATTCAGTTGAGT CACAGGAGA
piR034406	RT	CTCAACTGGTGTCGTGGAGTCGGCAATTCAGTTGAGA CGTCTAAAC
piR012524	RT	CTCAACTGGTGTCGTGGAGTCGGCAATTCAGTTGAGT CTATTCAGC

piR028356	RT	CTCAACTGGTGTCGTGGAGTCGGCAATTCAGTTGAGT CTATTCAGA
piR022629	RT	CTCAACTGGTGTCGTGGAGTCGGCAATTCAGTTGAGC TACTCAGCT
U6	RT	CTCAACTGGTGTCGTGGAGTCGGCAATTCAGTTGAGA AAATATG

Supplementary Table S7. Bisulfite PCR primers.

**Mouse**	**F/R**	**Sequence (5'-3')**
	***Gnas* promoter chr2: 174,297,264-174,297,575**	
Gnas	F	TTGTTTGAGTGTGGGGTTTTTA
	R	CTTTAAAAACTTTTCTATTCTCTC
	***Lhcgr* intergenic chr17:88,854,013-88,854,183**	
Lhcgr	F	GTTTTTTTGTGTGTTAGAGGTAT
	R	CTCTAACCTCTTTATTAAACATC
